# A Novel Machine Learning Algorithm for Creating Risk-Adjusted Payment Formulas

**DOI:** 10.1001/jamahealthforum.2024.0625

**Published:** 2024-04-19

**Authors:** Corinne Andriola, Randall P. Ellis, Jeffrey J. Siracuse, Alex Hoagland, Tzu-Chun Kuo, Heather E. Hsu, Allan Walkey, Karen E. Lasser, Arlene S. Ash

**Affiliations:** 1 Center for Innovation in Population Health, College of Public Health, University of Kentucky, Lexington; 2 Department of Economics, Boston University, Boston, Massachusetts; 3 Division of Vascular and Endovascular Surgery, Boston Medical Center, Boston University Chobanian and Avedisian School of Medicine, Boston, Massachusetts; 4 Institute of Health Policy, Management and Evaluation, University of Toronto, Toronto, Ontario, Canada; 5 BMC HealthNet Plan, Boston, Massachusetts; 6 Department of Pediatrics, Boston University Chobanian and Avedisian School of Medicine, Boston, Massachusetts; 7 Department of Medicine, University of Massachusetts Chan Medical School, Worcester; 8 Section of General Internal Medicine, Department of Medicine, Boston University Chobanian and Avedisian School of Medicine, Boston, Massachusetts; 9 Community Health Sciences, Boston University School of Public Health, Boston, Massachusetts; 10 Boston Medical Center, Boston, Massachusetts; 11 Senior Editor, *JAMA*; 12 Department of Population and Quantitative Health Sciences, University of Massachusetts Chan Medical School, Worcester

## Abstract

**Importance:**

Models predicting health care spending and other outcomes from administrative records are widely used to manage and pay for health care, despite well-documented deficiencies. New methods are needed that can incorporate more than 70 000 diagnoses without creating undesirable coding incentives.

**Objective:**

To develop a machine learning (ML) algorithm, building on Diagnostic Item (DXI) categories and Diagnostic Cost Group (DCG) methods, that automates development of clinically credible and transparent predictive models for policymakers and clinicians.

**Design, Setting, and Participants:**

DXIs were organized into disease hierarchies and assigned an Appropriateness to Include (ATI) score to reflect vagueness and gameability concerns. A novel automated DCG algorithm iteratively assigned DXIs in 1 or more disease hierarchies to DCGs, identifying sets of DXIs with the largest regression coefficient as dominant; presence of a previously identified dominating DXI removed lower-ranked ones before the next iteration. The Merative MarketScan Commercial Claims and Encounters Database for commercial health insurance enrollees 64 years and younger was used. Data from January 2016 through December 2018 were randomly split 90% to 10% for model development and validation, respectively. Deidentified claims and enrollment data were delivered by Merative the following November in each calendar year and analyzed from November 2020 to January 2024.

**Main Outcome and Measures:**

Concurrent top-coded total health care cost. Model performance was assessed using validation sample weighted least-squares regression, mean absolute errors, and mean errors for rare and common diagnoses.

**Results:**

This study included 35 245 586 commercial health insurance enrollees 64 years and younger (65 901 460 person-years) and relied on 19 clinicians who provided reviews in the base model. The algorithm implemented 218 clinician-specified hierarchies compared with the US Department of Health and Human Services (HHS) hierarchical condition category (HCC) model’s 64 hierarchies. The base model that dropped vague and gameable DXIs reduced the number of parameters by 80% (1624 of 3150), achieved an *R*^2^ of 0.535, and kept mean predicted spending within 12% ($3843 of $31 313) of actual spending for the 3% of people with rare diseases. In contrast, the HHS HCC model had an *R*^2^ of 0.428 and underpaid this group by 33% ($10 354 of $31 313).

**Conclusions and Relevance:**

In this study, by automating DXI clustering within clinically specified hierarchies, this algorithm built clinically interpretable risk models in large datasets while addressing diagnostic vagueness and gameability concerns.

## Key Points

**Question:**

Can a machine learning algorithm be used to produce risk adjustment models that respect clinical logic, address upcoding incentives, and predict costs better, especially for uncommon diseases, than the US Department of Health and Human Services (HHS) 2020 Affordable Care Act Marketplace hierarchical condition category (HCC) model?

**Findings:**

In this economic evaluation, the Diagnostic Cost Group (DCG) machine learning algorithm used clinician-specified hierarchies to predict top-coded total annual health care spending. The DCG algorithm achieved a higher *R*^2^ value despite excluding vague and gameable diagnoses and dramatically reduced HHS-HCC underpayments for rare conditions.

**Meaning:**

In this study, the DCG algorithm addressed gaming concerns and predicted costs better than the HHS-HCC model.

## Teaser

This economic evaluation creates and evaluates a machine learning algorithm for use in large datasets that can automate development of risk adjustment models.

## Introduction

Diagnosis-based risk adjustment formulas are widely used for health plan capitation, performance assessment, research-oriented severity adjustment, and value-based incentive payments and penalties. In the US, risk adjustment is used in Medicare Advantage, Affordable Care Act marketplaces, Medicare Part D prescription drug benefit programs, and state Medicaid managed care payment formulas, collectively accounting for more than $850 billion in 2021. Similar diagnosis-based predictive models are used by private and public organizations both nationally and internationally.

The hierarchical condition categories (HCCs) used by the US Centers for Medicare & Medicaid Services (CMS)^1,2^ and the US Department of Health and Human Services (HHS) for Medicare and Affordable Care Act Marketplace enrollees,^3,4^ respectively, have changed minimally in more than 2 decades despite huge increases in the diagnostic specificity of claims data since 2015.^5^ Numerous articles have articulated concerns with HCC models,^6,7,8,9^ including their failure to take advantage of richer diagnostic information, larger datasets, faster computers, and improved machine learning (ML) methods.^10^ Recent policy research has focused on their vulnerability to diagnostic upcoding, gaming, and fraud.^11,12^ CMS and others have recently reiterated and relied on the 10 original principles underlying the HCC models to change hierarchies to reduce payment formula vulnerability to gaming and improve fairness for patients and health plans.^13,14^ Despite ML’s enormous potential, its lack of transparency is concerning.^14^

In this study, we address all these concerns with a novel ML algorithm that continues to follow the recently reaffirmed 10 principles^1,2,15^ (eTable 1 in Supplement 1). This article enhances the utility of the Diagnostic Items (DXIs) diagnostic clustering system^5^ and refines both HHS HCC Marketplace modeling logic and the original Diagnostic Cost Group (DCG) framework for predicting health care spending.^16^ To address gameability concerns, we asked physician panels to score each DXI as to how comfortable they felt with its diagnoses being allowed to affect payment. Also, to reduce rewards for coding proliferation, we asked them to create hierarchies that enable the model to reflect the dominance of some conditions over clinically related, but less serious, conditions. We then studied the performance of models to predict cost produced by the ML algorithm, taking advantage of how easily it handles large datasets, to evaluate trade-offs among model simplicity, vulnerability to gaming, and predictive power.

## Methods

### Data and Study Sample

We used data from 2016 to 2018 of the Merative (formerly IBM) MarketScan Commercial Claims and Encounters Database including 65 901 460 person-years of enrollees 64 years and younger and enrolled in noncapitated commercial insurance plans with medical, pharmacy, and behavioral health benefits. We randomly split the data 90% to 10% for model development and validation, respectively, and used weighted least-squares regression to predict (concurrent) total spending top-coded at $250 000 per person-year, weighting by fraction of the year eligible.^1,3,5^ The Boston University Institutional Review Board exempted this study of deidentified data from review and informed consent. This study followed the Standards for Reporting of Diagnostic Accuracy (STARD) reporting guideline.

### Model Development

**Figure 1.**
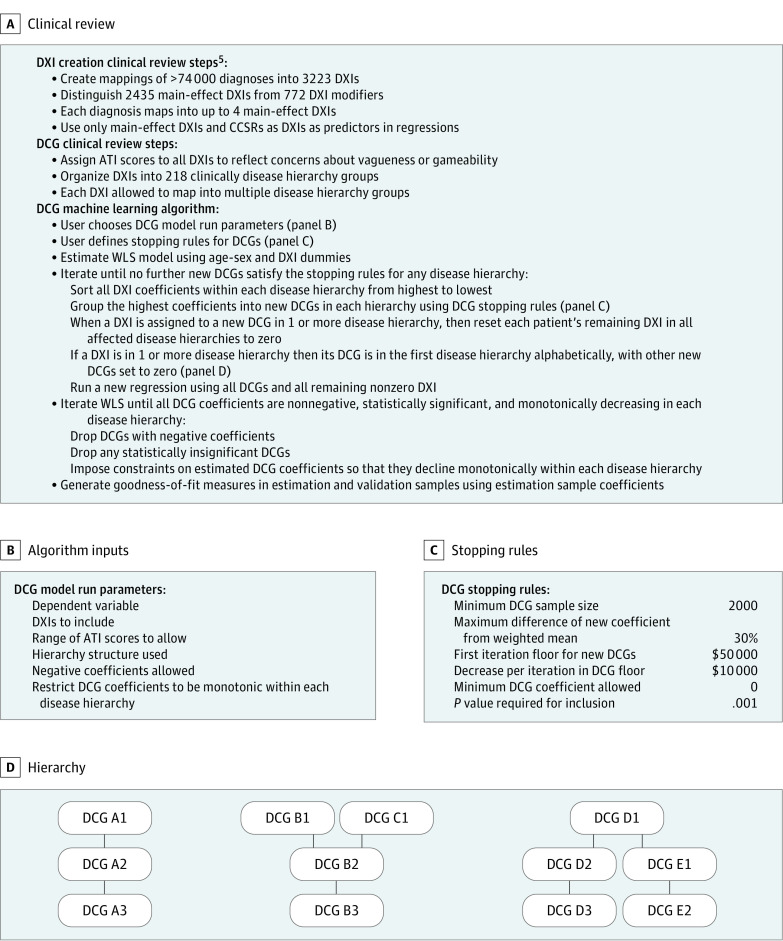
Overview of Diagnostic Item (DXI) and Diagnostic Cost Group (DCG) Clinical and Machine Learning Algorithm Steps ATI indicates Appropriateness to Include; CCSR, Clinical Classification Software Revised; WLS, weighted least squares.

The project had 2 stages (Figure 1A). First, we used clinical judgment to organize diagnosis codes into groups and hierarchies to be used as model building blocks. Then, we developed, implemented, and evaluated a novel ML algorithm to automate and empirically organize these groups and hierarchies into clusters for variable selection and prediction.

### Stage 1: Clinical Review

#### Clustering Diagnoses Into DXIs

We had previously used expert clinician review to assign all 72 000 US *International Statistical Classification of Diseases, Tenth Revision, Clinical Modification *(*ICD*-*10*-*CM*) billable diagnosis codes and their unbillable root codes to at least 1 and up to 7 diagnostic clusters (ie, DXIs).^5^ We grouped diagnoses into the same DXI when they were clinically related and had similar costs and clinical implications (for example, pneumonia due to organism A vs organism B).^5^ We further categorized DXIs as either main-effect DXIs that describe distinct diagnoses or modifier DXIs that provide further specificity concerning location (eg, left, right, or bilateral) or timeline (eg, acute or chronic and initial, subsequent, or sequela).^5^ In addition to DXI assignments, we also grouped diagnoses based on the Clinical Classification Software Revised (CCSR)^17^ framework. Both DXI and CCSR classifications are many-to-many mappings. Because main-effect DXIs and CCSRs are included in the same way in all analyses here, in the rest of this article, DXIs refer to the set containing all categories from either classification.

#### Assigning Appropriateness to Include Scores to DXIs

Expanding on prior risk adjustment methods,^16,18,19^ small groups of clinicians and health policy experts familiar with *ICD*-*10*-*CM* coding assigned each DXI an Appropriateness to Include (ATI) score, rating its attractiveness for inclusion in payment models. Scores reflect concerns about vagueness and code manipulability (such as the desire to not allow plan payments to rise simply because vague codes are piled on) and ranged from 0 (no concerns) to 5 (serious concerns). eTable 2 in Supplement 1 displays scoring instructions. Scoring was anchored by telling reviewers to think of an ATI of 4 as a plausible threshold for model exclusion. Most DXIs in 3 of *ICD*-*10*-*CM*’s 21 chapters (“Symptoms, Signs and Abnormal Findings,” “External Causes of Morbidity,” and “Factors Influencing Health Status”) received ATIs of 5 and thus were excluded from the base model, which only included DXIs with ATIs of 3 or less.

#### Organizing the DXIs Into Clinical Disease Hierarchies

The clinicians gave each DXI, disease hierarchy, and DCG variable a helpful label reflecting clinical or cost features and mapped DXIs to disease hierarchy groups. In contrast to the CMS HCC^2^ and HHS HCC^3^ approaches, each DXI could map to any of 218 disease hierarchy groups, some of which cut across disease chapters. For example, the DXI containing the hypertensive retinopathy diagnosis mapped into both the circulatory hypertension and eye vitreoretinal disease hierarchy groups.

### Stage 2: ML Algorithm

We then developed, implemented, and evaluated an ML algorithm that uses several tuning parameters, the DXIs, ATI scores, and disease hierarchy groupings to produce predictive models (Figure 1A).

### DCGs

The central contribution of the ML algorithm was to create DCGs that are ordered clusters of DXIs with similar cost implications; DXIs in higher-cost DCGs cause the model to ignore lower-cost DXIs when they are in the same hierarchy (that is, when they are clinically related). Because DXIs can belong to multiple disease hierarchies, a single DXI can knock out (or override) less serious conditions in multiple hierarchies; this enables hierarchy logs (straight lines), branches (mergers), and roots (splits), as in Figure 1D.^1^ For example, an asthma diagnosis overrides cough but not diabetes, and type 1 diabetes overrides both type 2 diabetes and unspecified diabetes. The ML algorithm parallels the methods used to develop the HHS HCC models^3^ but in a way that is automated, transparent, and replicable.

### DCG ML Algorithm

Figure 1 summarizes the DCG ML algorithm steps that are described in more detail in the eMethods in Supplement 1. Here, we focus on predicting top-coded total health care spending, but the algorithm is generic. The algorithm focuses on finding sets of variables with the same incremental (or marginal) contribution to the outcome rather than on finding subsets of the data with similar outcome averages. Our approach differs from regression tree models and other ML models that sequentially split a sample to maximize between-group variance at each step but never look back when considering new splits. Regression trees favor common over rare conditions and ignore rare conditions that are split across many categories. In contrast, we impose hierarchies on sets of coefficients within an additive regression framework, allowing information from multiple hierarchies to help explain outcomes and identify the incremental costs of both common and rare conditions. As is true in the existing HHS HCC system, the sum of the coefficients of each individual’s DCG across all disease hierarchy groups is an individual’s predicted cost. For example, the predicted cost of a patient with both asthma and diabetes includes the sum of the incremental cost of these 2 DCG coefficients, not the average cost of only 1 of them. Age and sex dummies also contribute to predicted cost.

The DCG algorithm requires user-specified inputs (Figure 1B) characterizing the dependent and independent variables and key elements of model structure; there must also be user-specified stopping rules (Figure 1C) based on minimum sample sizes, levels of statistical significance, maximum heterogeneity allowed within an estimated group, and 3 DCG floor parameters that affect the dispersion of DCGs across hierarchies. For example, the initial DCG floor parameter of $50 000 ensures that across all disease hierarchy groups, only DCGs costing more than $50 000 are created on the first iteration, and the $10 000 interval means that the second iteration considers DCGs costing at least $40 000.

Model estimation was done in 2 sets of iterations. First, the model iterates to sequentially find the highest coefficient DXIs in each disease hierarchy group, which are grouped into DCGs before assigning lower-coefficient DXIs to lower-cost DCGs. When a patient is assigned to a DCG, all unassigned (and hence, lower-coefficient) DXIs within the same hierarchy are reset to zero; each enrollee is assigned to at most 1 DCG per disease hierarchy group. The spillover effect of DXIs falling in multiple hierarchies is described in eFigure 2 in Supplement 1. Once all DCG stopping rules are satisfied, the algorithm initiates a further round of iterations to improve face validity and model parsimony, first by dropping DCGs with negative or statistically insignificant coefficients and then by constraining adjacent DCGs to have the same coefficient when they are not monotonically decreasing within each disease hierarchy for each higher-numbered DCG.

### Statistical Analysis

After fitting each ML model in the development data, its performance was evaluated in the validation data. The algorithm calculates *R*^2^ values, mean absolute errors (MAEs), and a novel measure examining predictive accuracy for all enrollees with at least 1 rare diagnosis. For all models, we display the average residual for the set of enrollees with at least 1 disease more rare than 1 in 10 000, and for 5 key model specifications, we show average residuals across the full range of each patient’s rarest diagnosis. Model parsimony was measured by the number of model parameters used. All analyses were conducted using SAS version 9.4 (SAS Institute).

## Results

This study included 35 245 586 commercial health insurance enrollees 64 years and younger (65 901 460 person-years) and 19 clinicians who provided reviews in the base model. We estimated our base case DXI DCG ML model (Figure 1B) on the estimation sample of 59 million person-years using DCG stopping rules (Figure 1C) applied to 218 disease hierarchies, which started out with 2435 main-effects DXIs, 537 CCSR categories, and 30 age-sex dummies. Hierarchies had diverse log, tree branch, and root structures, as in Figure 1D. After removing DXIs with ATI scores greater than 3, DXIs in the *ICD*-*10*-*CM* “External Causes of Morbidity,” and “Factors Influencing Health Status” chapters, and colinear or nearly perfectly colinear DXIs, our base model started iterating with 2366 parameters. eTable 5 in Supplement 1 displays the final DCG regression results.

The Table shows model performance using the validation sample, which is shown in eTable 3 in Supplement 1 to closely mimic the development sample. The base DCG model had an *R*^2^ of 0.535 (MAE, $4114). The base DCG model performed notably better than using Charlson Comorbidity Index^20^ variables as predictors (*R*^2^ = 0.227; MAE, $6116) or using diagnostic categories from the HHS HCC model^21^ (*R*^2^ = 0.428; MAE, $5227). As expected, the base DCG model *R*^2^ value was slightly lower than the additive DXI plus CCSR model,^5^ which includes additional parameters at the expense of gameability and coding proliferation. The base DCG model used 624 parameters, a reduction of 80% relative to the 3150 in the full DXI additive model.

**Table.** Sensitivity Analysis: Validation Sample Measures of Alternative Specifications

Model^a^	*R* ^2^	MAE, $	Parameters, No.	Rare disease mean error, $^b^
Base DCG model^c^^,^^d^	0.535	4114	624	−73
Alternative model structures^d^				
CCI^e^	0.227	6116	48	3055
HHS HCC Marketplace using hierarchies	0.428	5227	166	1927
CCSR additive model	0.539	4140	567	−114
DXI^d^ plus CCSR additive model^f^	0.589	3786	2929	−83
Disease chapters additive model	0.201	6226	52	556
Hierarchy groups additive model	0.447	4756	249	−52
ATI score^g^				
0	0.469	4520	445	610
<2	0.503	4313	526	296
<3	0.526	4151	619	−4
<4 (Base without forcing monotonicity)	0.535	4113	661	−71
<5	0.536	4134	667	−115
All values	0.539	4112	683	−109
Alternative information sets^d^				
Including 2 *ICD*-*10*-*CM* chapters excluded in base model^h^	0.568	3910	710	−139
Allow negative and insignificant coefficients	0.534	4114	672	−74
No exclusions imposed within hierarchies	0.541	4071	687	−87
Single hierarchy for each chapter	0.495	4339	202	−26
Single hierarchy	0.315	5031	28	898
Base model using CCSR variables only	0.461	4514	248	212
Base model using DXI variables only	0.524	4170	676	22

Abbreviations: ATI, Appropriateness to Include; CCI, Charlson Comorbidity Index; CCSR, Clinical Classifications Software Refined; DCG, Diagnostic Cost Group; DXI, Diagnostic Item; HCC, hierarchical condition category; HHS, US Department of Health and Human Services; *ICD*-*10*-*CM*, *International Statistical Classification of Diseases, Tenth Revision, Clinical Modification*; MAE, mean absolute error.

^a^ All models included 30 age-sex dummy variables.

^b^ Mean residual of enrollee-years with any diagnosis rarer than 100 per million.

^c^ Monotonicity was imposed for the final base model by restricting DCG coefficients to be decreasing in each disease hierarchy.

^d^ Models retained only DXIs with ATI scores less than 4.

^e^ We included 18 dummy variables as defined by Quan et al.^20^

^f^ This DXI plus CCSR additive model was the base model used by Ellis et al.^5^

^g^ Scored from 0 (least gameability concerns) to 5 (most gameability concerns).

^h^ Includes the “External Causes of Morbidity,” and “Factors Influencing Health Status” *ICD*-*10*-*CM* chapters.

### ATI Scores

**Figure 2.**
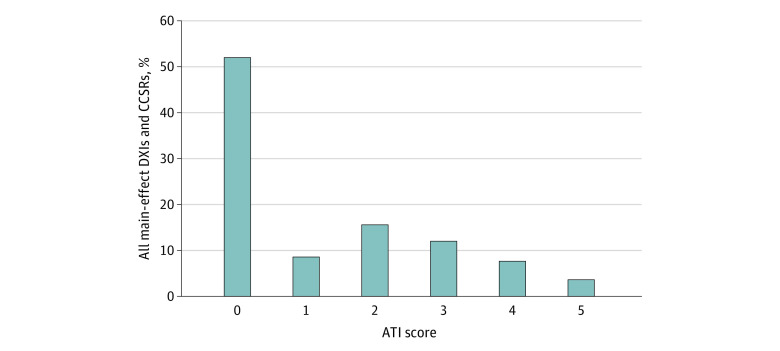
Distribution of Appropriateness to Include (ATI) Scores in Diagnostic Item (DXI) Main Effects and Clinical Classification Software Revised (CCSR) Classifications Percentages are calculated as the fraction of all main-effect DXIs and CCSRs.

Figure 2 shows the distribution of ATI scores. Moderate to serious concerns about using DXIs with an ATI score of 4 or 5 were flagged for 362 of 3150 DXIs (12%). Importantly, model performance was largely unaffected by ignoring the most concerning groups; the *R*^2^ changed from 0.539 when all diagnoses were included to 0.535 for the base model (Table). In contrast, dropping all 1508 DXIs (48%) that raised any concerns about inclusion (ATIs greater than 0) notably reduced the *R*^2^ to 0.469.

### Detailed DCG Results

**Figure 3.**
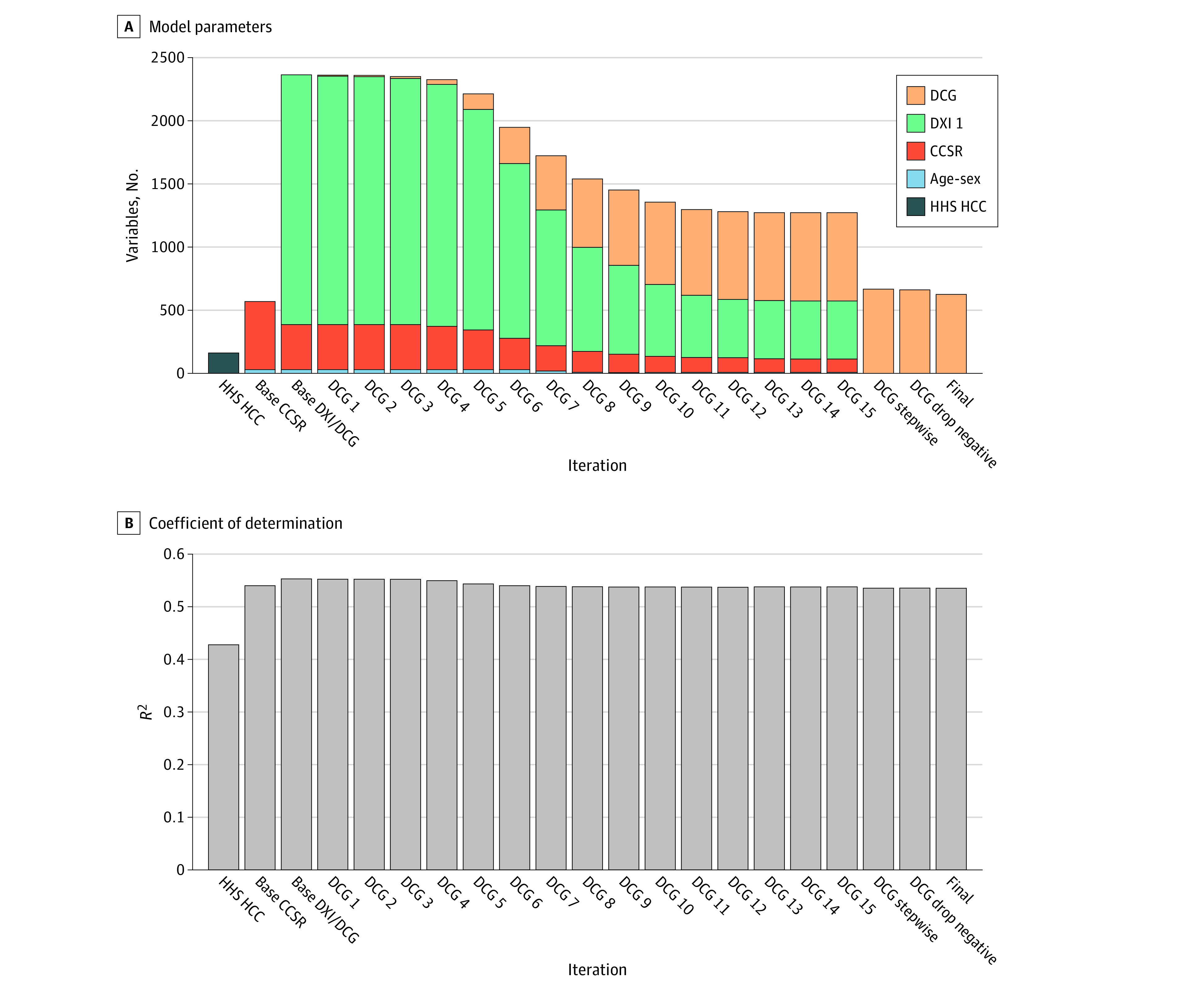
Model Parameter Counts and *R*^2^ across Diagnostic Cost Group (DCG) Iterations for the Base Model The US Department of Health and Human Services (HHS) hierarchical condition category (HCC) model used the combined set of HHS HCCs included in the adult, child, and infant models in a single regression. The Clinical Classification Software Revised (CCSR) model used weighted least squares on all 538 observed CCSR categories, while the base Diagnostic Item (DXI) model used main-effect DXIs and CCSRs. As DCGs were created, DXIs assigned to them were dropped from the model. After all DCGs were found, the DCG stepwise iteration estimated a stepwise regression that omitted all remaining DXI variables not assigned to DCGs and included only statistically significant and nonnegative DCGs. The final run constrained coefficients to be monotonically decreasing within disease hierarchies. All models included 30 age-sex dummy variables.

Detailed outcomes across iterations and final regression coefficients are illustrated in Figure 3 and eTables 4 and 5 in Supplement 1. eFigures 1 to 4 in Supplement 1 also include full detail for 40 disease hierarchies in 3 *ICD*-*10*-*CM* chapters: circulatory system; endocrine, nutritional, and metabolic; and injuries, poisoning, and other external causes.

Figure 3A reveals how variables are added and then dropped in the ML algorithm and also contrasts the model with the simple additive HHS HCC and CCSR additive models. The DCG ML algorithm required 15 iterations to identify all DCGs and then 4 more iterations were required to ensure nonnegativity, enforce tighter statistical significance, and impose monotonicity so that DCG coefficients within hierarchies always decline. Although a fairly large number of DXIs were initially considered, the base model has many fewer parameters, with only a modest reduction in *R*^2^.

Figure 3B provides development sample adjusted *R*^2^ values across a range of DCG iterations, highlighting that simplifying the model by using a single coefficient to apply to similarly costed conditions that are clinically related only modestly reduced model fit (*R*^2^ from 0.553 to 0.536). The validation sample *R*^2^ for the base DCG model matches that of the development sample at 0.535. eTable 5 in Supplement 1 shows all DCG coefficients.

### Rare Diagnoses

**Figure 4.**
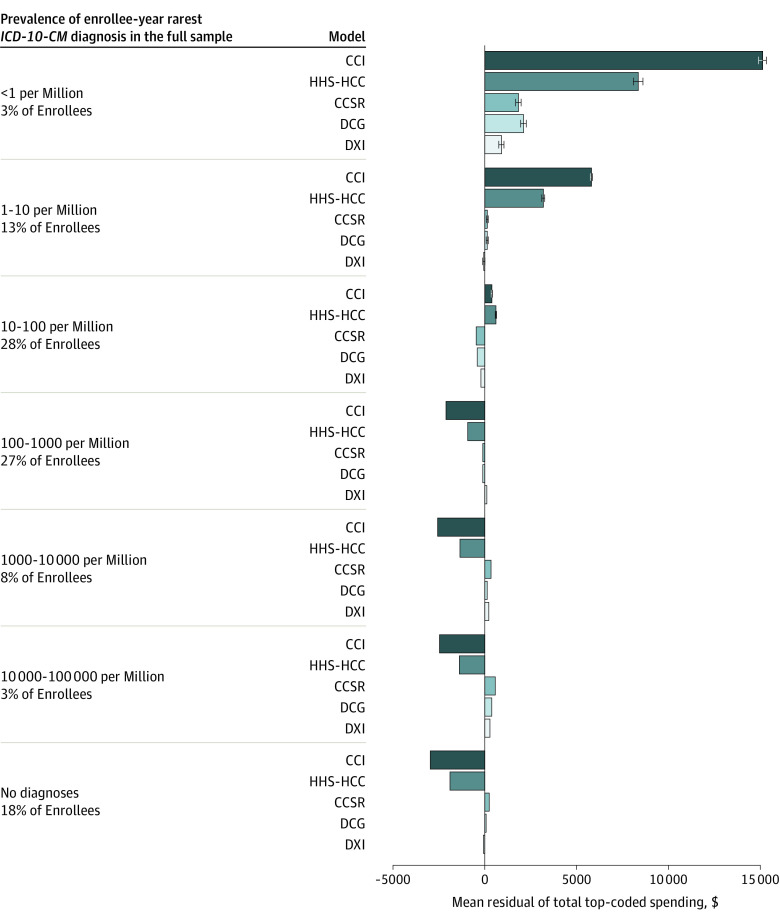
Mean Residuals of Total Spending in the Validation Sample Top-Coded at $250 000 for 5 Models by Frequency of Enrollee-Year Rarest Diagnosis All models include age-sex dummy variables. We calculated enrollee-weighted mean residuals in the validation sample using the binned frequencies of diagnoses in the full sample, with frequency intervals determined by powers of 10 per million. Plot whiskers indicate 95% CIs, corrected for clustering at the patient level. CCI indicates Charlson Comorbidity Index^20^; CCSR, Clinical Classifications Software Refined; DCG, Diagnostic Cost Group; DXI, Diagnostic Item; HCG, hierarchical condition category; HHS, US Department of Health and Human Services; *ICD*-*10*-*CM*, *International Statistical Classification of Diseases, Tenth Revision, Clinical Modification*.

Figure 4 presents the sample percentiles and mean residuals for groups of enrollees distinguished by the rarity of their most uncommon diagnosis, calculated for 7 bins of enrollees with probabilities changing by powers of 10. This measure used the rarity of individual *ICD*-*10*-*CM* codes, not groups such as DXIs, and hence is neutral across models. Rates of at least 1 rare condition were much more common than might be expected, with 988 034 of 6.6 million enrollees having at least 1 diagnosis rarer than 10 per million. Enrollees with at least 1 low-frequency diagnosis were greatly underpredicted by the HHS HCC model and Charlson Comorbidity Index model; only the CCSR, DXI, and DCG models improved this measure of performance. Enrollees with no diagnoses were overpaid by more than $2000 in the HHS HCC model. As previously described,^5^ the additive DXI model substantially outperformed the HHS HCC model across the spectrum of common to rare diagnoses. Figure 4 illustrates that although DXI accuracy worsened when parsimony and incentives were enforced, DCGs still performed well.

### Sensitivity Analysis

We conducted a sensitivity analysis by estimating models under different assumptions to evaluate the trade-offs between incentives and predictive power and the robustness of the results across assumptions and various stopping rules. The DCG model was largely insensitive to stopping rule choices (eTable 4 in Supplement 1).

We also explored sensitivity to changing the model hierarchy structures and diagnostic information used (Table). The base model outperformed all DCG variants except for 2 felt to have worse incentives: one variant included DXIs from *ICD*-*10*-*CM* chapters (“External Causes of Morbidity and Mortality” and “Factors Influencing Health Status and Contact With Health Services”), and the other did not ignore DXIs already assigned to DCGs and hence was more additive. The first of these was informative in that some diagnoses reflected social determinants of health dimensions that may affect health care costs; however, there was concern about using these diagnoses because of their poor quality in existing claims data.^8,22^

Finally, our use of multiple hierarchies within each *ICD*-*10*-*CM* chapter provided significant gains. Alternative models that used additive disease hierarchy dummies (equivalent to 1 DCG per disease hierarchy) or allowed only 1 hierarchy per chapter or 1 hierarchy across all chapters (as done in the original DCG model by Ash et al^16^) performed less well than the base DCG model.

## Discussion

This study used DXIs to organize information for risk adjustment prediction, leveraging the diagnostic detail of *ICD*-*10*-*CM* codes and incorporating many refinements proposed for the World Health Organization’s recently introduced *ICD*-*11* codes just beginning to be evaluated for the US.^23^ In this study, we built on the existing DXI system by automating methods to aggregate information into DCGs for prediction. This simplification resulted in a very small loss of predictive power yet facilitated estimation on smaller samples and reduced model vulnerability to upcoding.

The DXI DCG system relies on the use of hierarchies, as carefully emphasized in both the earliest and most recent list of principles for developing risk adjustment models.^1,15^ We have added 2 more principles to our list included in eTable 2 in Supplement 1: principle 11 specified that models should do well even on sets of rare diagnoses and principle 12 specified that parsimonious models with fewer parameters are preferred.

The automated DCG algorithm is a multiple-hierarchy extension of the original DCG model developed by Ash and colleagues.^16^ Automating the mapping of DXIs to DCGs enabled us to explore models with various hierarchical and nonhierarchical structures and exclusion thresholds (ATI score cutoffs). The algorithm can be used to build and evaluate models predicting other outcomes and for different purposes. The DXIs have already been shown to be highly predictive of utilization and quality outcomes.^5^ Assigning DXIs to multiple hierarchies allows for rich hierarchical structures (Figure 1D) in which predictions focus on the most consequential manifestations of each disease process.

Our approach differs from the usual ML algorithms that estimate risk adjustment models, which draw repeated samples of less than 1 million and fit models with prespecified sets of predictors.^10,24,25^ While repeated sampling improves parsimony and tractability, such methods cannot accurately predict the costs of expensive rare conditions. All other risk adjustment models that we examined greatly underpay for enrollees with rare medical conditions.

A 2021 review of state-of-the-art predictive modeling^26^ found that people with multiple chronic conditions had the highest health care spending and service utilization. Although many ML methods work well on healthy populations or when morbidity measures cover only a few diseases, ML branching algorithms (eg, regression trees and random forests) perform poorly in large samples when multimorbidity drives the outcome. These approaches cannot see the cumulative effect of multiple diseases in a single person.

The automated DCG algorithm makes it easy to model trade-offs among efficiency incentives, fairness concerns, feasibility, and simplicity. While its model structure is similar to the HHS HCC model, it better differentiates rare, higher-cost conditions and has greater overall predictive power. The DCG ML algorithm models are linear in predictors and can ignore diagnoses due to vagueness, inconsistent use, or gameability. Other attractive features are hierarchy restrictions that ignore lower-ranked diagnoses when in the presence of clinically related, higher-ranked diagnoses and final models that avoid negative predictions. Sensitivity analyses using physician ratings to exclude vague and gameable DXIs suggest that many more diagnoses could be permitted in models than the 14% of diagnosis codes that the HHS HCC 2022 model includes.

### Limitations

This study has limitations. This study applied its algorithm only to concurrent total spending in a commercially insured population; future work could study other outcomes, periods, populations, and uses, such as the prospective models used by Medicare. We predicted only total spending top-coded at $250 000, which we believe is better at identifying key explanatory variables, but for implementation models predicting un–top-coded covered spending may be preferred. Additionally, the model did not use prescription drug information or other variables including measures of social determinants of health as predictors. The newly created hierarchies, ATI scores, nonnegativity constraints, and enforced monotonicity across multiple hierarchies may prove useful in other applications but have not yet been evaluated.

## Conclusions

This study advances the utility of the DXI system in important ways. For each DXI, an ATI score was developed and used it to select DXIs to use as predictors in a framework where DXIs are mapped into 1 or more of 218 newly constructed and clinically driven hierarchies that focus payments on the most serious manifestations of each disease process. With little loss in predictive power, the ML algorithm identified DCGs that reduced the number of parameters needed by 80% and reduced model sensitivity to upcoding compared with simple additive models. The DCG algorithm creates additive regression coefficients on sorted clusters of diseases (DCGs) that prioritize recognition of the most serious (ie, costly) diseases within each hierarchy while ignoring less serious diseases for the same enrollee. Finally, the model, developed on a large dataset, can reliably price even rare diseases, avoiding serious underpayments even for the 3% of people who have at least 1 diagnosis as rare as 1 in 1 million.

## Notes

Supplement 1. **eMethods.** DCG Algorithm Details **eFigure 1.** Additive DXI Model Circulatory Coefficients Organized into 13 Hierarchies **eFigure 2.** DCG Model Coefficients for Sets of Circulatory DXIs, DCG Version 1.1 **eFigure 3.** DCG Model Coefficients for Sets of Endocrine, Nutritional, and Metabolic DXIs, DCG Version 1.1 **eFigure 4.** DCG Model Coefficients for Sets of INJ DXIs, DCG Version 1.1 **eTable 1.** Modeling Principles Used in This Project as Expanded From Ash et al (2000)^6^ in CMS-CIOO (2021)^7^ **eTable 2.** Scale Used by Clinicians to Assign Appropriateness to Include Scores **eTable 3.** Summary Statistics From Development and Validation Samples **eTable 4.** Sensitivity Analysis: Validation Sample Measures of Alternative Model Tuning Parameters **eTable 5.** Coefficients From DCG WLS Regression Best Incentives DCG ML Model
eReferences.

Supplement 2. Data Sharing Statement
