# Supplementary material for: A Novel Machine Learning Algorithm for Creating Risk-Adjusted Payment Formulas
Source: JAMA Health Forum. 2024 Apr 19;5(4):e240625. doi: 10.1001/jamahealthforum.2024.0625 (PMC11065160; doi:10.1001/jamahealthforum.2024.0625)
Supplement: Supplement 1. — eMethods. DCG Algorithm Details eFigure 1. Additive DXI Model Circulatory Coefficients Organized into 13 Hierarchies eFigure 2. DCG Model Coefficients for Sets of Circulatory DXIs, DCG Version 1.1 eFigure 3. DCG Model Coefficients for Sets of Endocrine, Nutritional, and Metabolic DXIs, DCG Version 1.1 eFigure 4. DCG Model Coefficients for Sets of INJ DXIs, DCG Version 1.1 eTable 1. Modeling Principles Used in This Project as Expanded From Ash et al (2000)6 in CMS-CIOO (2021)7 eTable 2. Scale Used by Clinicians to Assign Appropriateness to Include Scores eTable 3. Summary Statistics From Development and Validation Samples eTable 4. Sensitivity Analysis: Validation Sample Measures of Alternative Model Tuning Parameters eTable 5. Coefficients From DCG WLS Regression Best Incentives DCG ML Model eReferences. [file jamahealthforum-e240625-s001.pdf]

Supplemental Online Content

Andriola C, Ellis RP, Siracuse JJ, et al. A novel machine learning algorithm for creating risk-adjusted payment formulas. *JAMA Health Forum.* 2024;5(4):e240625. doi:10.1001/jamahealthforum.2024.0625

eMethods. DCG Algorithm Details

eFigure 1. Additive DXI Model Circulatory Coefficients Organized into 13 Hierarchies

eFigure 2. DCG Model Coefficients for Sets of Circulatory DXIs, DCG Version 1.1

eFigure 3. DCG Model Coefficients for Sets of Endocrine, Nutritional, and Metabolic DXIs, DCG Version 1.1

eFigure 4. DCG Model Coefficients for Sets of INJ DXIs, DCG Version 1.1

eTable 1. Modeling Principles Used in This Project as Expanded From Ash et al (2000)[6] in CMS-CIOO (2021)[7]

eTable 2. Scale Used by Clinicians to Assign Appropriateness to Include Scores

eTable 3. Summary Statistics From Development and Validation Samples

eTable 4. Sensitivity Analysis: Validation Sample Measures of Alternative Model Tuning Parameters

eTable 5. Coefficients From DCG WLS Regression Best Incentives DCG ML Model

eReferences.

This supplementary material has been provided by the authors to give readers additional information about their work.

© 2024 Andriola C et al. *JAMA Health Forum*.

eMethods. DCG Algorithm Details

As described in the main text, a major goal of this project was to design a customized machine learning algorithm that efficiently aggregates DXIs into DCGs, and orders these DCG sets of DXIs within hierarchies following pre-specified criteria. The algorithm is similar to regression tree, random forest, and other automated algorithms in that once model parameters and decision criteria are chosen, the computer identifies the clusters.[1] A key difference is that we impose a clinical-based decision structure on the DCG formation process, to boost clinical coherence and reduce the reward for redundant diagnostic coding. A second difference is that while regression trees and random forests split samples into groups based on outcome averages, we cluster DXIs into multiple groups based on the having similar coefficients for predicting the outcome variable (here, topcoded total spending) without regard to fit statistics.

Supplement Figure 1 illustrates how DXIs are assigned to mutually exclusive DCGs in our DCG ML algorithm for the simple case in which each DXI a_i belongs to a single HIER group. In this example, each set of DXIs A_i is iteratively assigned to four DCGs $\{A_1, A_2, A_3, A_4\}$, until there are no more DXIs to assign that are nonnegative or statistically significant. With a single hierarchy, each individual is uniquely assigned to a single DCG, that is, only people not in DCG A_1 can be assigned to DCG A_2, and so on. DXI assignment to DCGs need not be exhaustive: DXIs whose incremental contributions to cost are not significantly greater than 0 are not assigned to a DCG.

Supplement Figure 2 illustrates a more complex case with two HIER groups, A and B, and one DXI (X) is initially included in both HIER A and B. It is not known *a priori* which HIER that DXI X will be assigned to, but the algorithm assigns X to the first HIER for which X is eligible to join a DCG, in this case A_2. (In the case of a tie in this assignment, X is assigned to the first HIER group alphabetically.) For each person with a diagnosis assigned to DCG A_2, the algorithm then resets to zero all not-yet-assigned DXI in both HIER A and HIER B. In this way, when a first DCG coefficient is assigned to this DXI for this person, all consideration ends of their less serious DXIs in both HIER (A) and HIER (B).

Equation (1) summarizes the linear framework used in a linear DXI model. Because DXI items and CCSR categories enter analogously in all models, we simplify notation and call both types of variables simply DXIs. Let i index enrollee-years, a index for age and gender groups, and j index DXI items. Using outcome Y_i as the dependent variable, let A_i be the enrollee's age and gender, and DXI_i be arrays of DXI and CCSR disease categories for enrollee-year i; let α and β be the appropriately indexed unknown parameters to be estimated for each set of variables; and let ε_i be the error on the observed outcome Y_i, then the base DXI model[2] can be written as:

$$Y_i = A_{ia} * \alpha_a + DXI_{ij} * \beta_j + \varepsilon_i \qquad (1)$$

Previous work estimated DXI formula (1) using the same 59 million enrollee sample and found 2,282 statistically significant parameters with no evidence of overfitting. Three problems were identified in using DXIs for payment, benchmarking, or performance assessment. First, 373 of the DXI and CCSR parameters (12.2%) in the topcoded model of concurrent spending were negative, which is unattractive for practical payment models because it predicts negative spending for many individuals and lacks face validity. Second the large parameter (low parsimony) models were unattractive for re-estimation on smaller size samples that lack the power to estimate coefficients for relatively rare DXIs. ML algorithms popular in the literature[1,3] reveal that more sparsely parameterized models are often superior in smaller sample sizes. A third problem is that an additive DXI formula does not address coding incentives, and will reward coding proliferation by increasing payments whenever more diagnoses are added, even when newly added codes are already implied by diagnoses already present. Coding "Diabetes, Unspecified Type" should not be recognized when a more specific diagnosis such as "Diabetes, Type 1" is also available.

The DXI Diagnostic Cost Group (DCG) Algorithm

The DCG algorithm presented here differs from the original Ash et al.[4] DCG formulation and its CMS implementation[5] in 2000 in five respects. First, we flexibly screen out DXIs considered vague or highly gameability as captured by their ATI scores. Second, we allow for multiple hierarchies for each DXI. Third, we cluster diagnostic items according to the similarity of their regression coefficients rather than their average costs. Fourth, we use specified statistical criteria for grouping DXIs into DCGs.[6] Fifth, the estimation is algorithmic, not manual, enabling ML models to be efficiently estimated for diverse outcomes in new samples for a variety of purposes in a reasonable amount of time. Note that DXIs or CCSRs that were perfectly colinear with sets of other variables were assigned an ATI score of 6 and hence excluded automatically.

© 2024 Andriola C et al. *JAMA Health Forum*.

Because age*gender variables enter additively in the base model and also needed to be constrained to be non-negative, two hierarchies were also created for female and male and included in the estimation algorithm analogously to other DXIs. The final DCG model specification can be written compactly as in (2). Within each hierarchy *h* we are creating DCG groups of DXIs, indexed by *g*, where the highest coefficient DCG within h is DCG_{h1}, and higher indexed DCGs having smaller coefficients. Within each hierarchy a person in DCG g' cannot also be assigned to DCG g" when g' < g".

$$Y_i = \sum_h \sum_g \left[DCG_{hg} * \beta_{hg} \right] + \varepsilon_i \qquad (2)$$

For ease of interpretation, DCGs are given informative names reflecting the ICD10 disease chapter and hierarchy, and numbered sequentially with 1 being the highest cost DCG.

The DCG model is estimated by iteratively choosing sets of DXIs to assign to high coefficient DCGs before choosing lower coefficient DCGs overall and within each HIER, with iterations continuing until stopping rules are satisfied. At the first iteration, only DXIs with incremental costs above $50,000 are eligible to be assigned to DCGs, a lower bound that is successively lowered as lower coefficient DCGs are identified. No DXI can end up in more than one HEIR, negative coefficients are not allowed, and within a hierarchy a given person can only be assigned one DCG. Iterations continue creating DCGs until no further DCGs satisfying the stopping rules can be created. (This required 14 iterations in the Base case specification)

The DCG ML algorithm then continues a second type of iteration intended to eliminate statistically insignificant, negative, and non-monotonic DCGs within each HIER group. This second type of iteration proceeds very speedily since once the full set of cross products of all DCGs and the dependent variables is created, these steps can be done by simply imposing constraints on the DCG coefficients. Specifically, the model first performs a backwards stepwise weighted least squares (WLS) regression that dropped variable with p values greater 0.0001. The model then constrains any negative DCG coefficients β_{hg} to be zero, and finally, if within any hierarchy h, $\beta_{hg'} < \beta_{hg''}$ when g' < g", then we restrict these two coefficients on these two DCGs to be the same. These three steps were performed repeated until all three desirable features of coefficients were satisfied (four iterations in the Base model specification)

Because imposing monotonicity has no meaning in strictly additive models, and requires extra processing, we did not impose monotonicity on all models used for sensitivity analysis, but instead did it once at the end of all estimation for our Base model specification.

Stopping Rules for DCG Groups

The number of DCGs created within each hierarchy is not specified *a priori*, but instead is controlled by six modeling parameters. They (with their Base case settings) are:

1. The minimum sample size for each DCG (2,000),
2. The maximum percent difference allowed between the current weighted average coefficients in a DCG and the next coefficient considered (30%),
3. The statistical significance required to assign a DXI to a DCG (p<0.001)
4. Whether to assign DXI with negative coefficient weights to DCG (no),
5. The initial floor to the DCG average used ($50,000),
6. The decrease in the floor in each iteration ($10,000).

Each of these six parameter was varied for sensitivity analysis.

Once the above stopping DCG rules were all satisfied the stopping rules, all remaining DXIs were dropped, and a backwards stepwise regression with an even tighter inclusion criteria (p<0.0001) was estimated to exclude less significant DCGs and negative coefficient DCGs. This final step was repeated until all included DCGs have non-negative coefficients.

When an enrollee has a DXI that is assigned a DCG in any hierarchies, all of their other DXIs in that hierarchy are reset to zero. This also happens when an assigned DXI is assign to other hierarchies. An implication of this process is that sample sizes in some DXIs are very low when they are added to a DCG or excluded from the model. The inclusion of very rare diagnostic information in model predictions is unique to our algorithm and not a feature of any other payment algorithm with which we are familiar.

© 2024 Andriola C et al. *JAMA Health Forum*.

Supplement Figure 1. Flow Chart of Assignment of DXIs to DCGs in a Single Hierarchy A

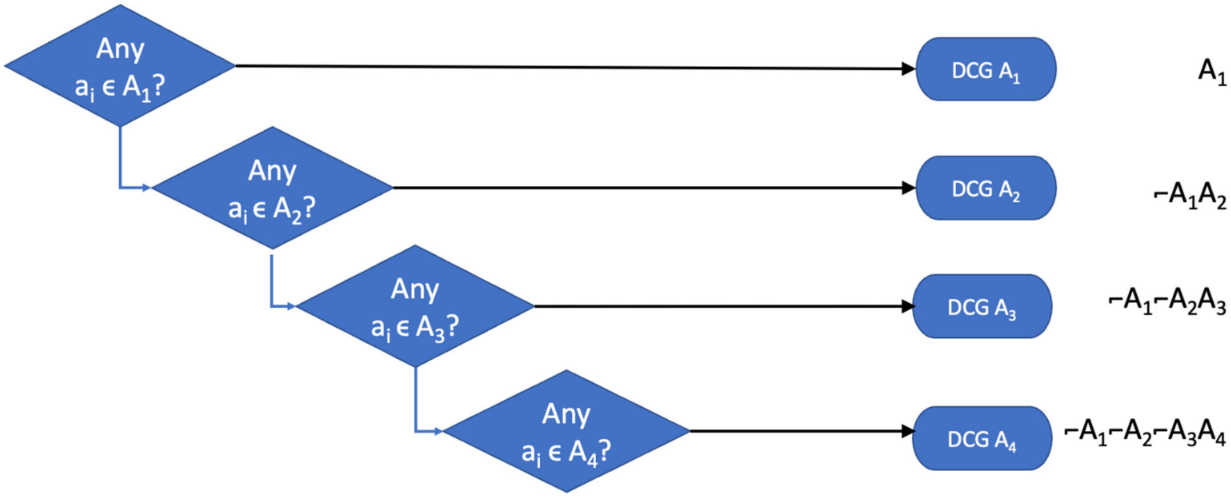

Notes: a_i are individual diagnostic items. A_i are sets of DXIs. ⌐ means not. DXI is a Diagnostic Item, and DCG is a Diagnostic Cost Group.

© 2024 Andriola C et al. *JAMA Health Forum*.

Supplement Figure 2. Flow Chart of Hypothetical DCG Algorithm Assignment When DXI_X Maps to Both Hierarchies A and B

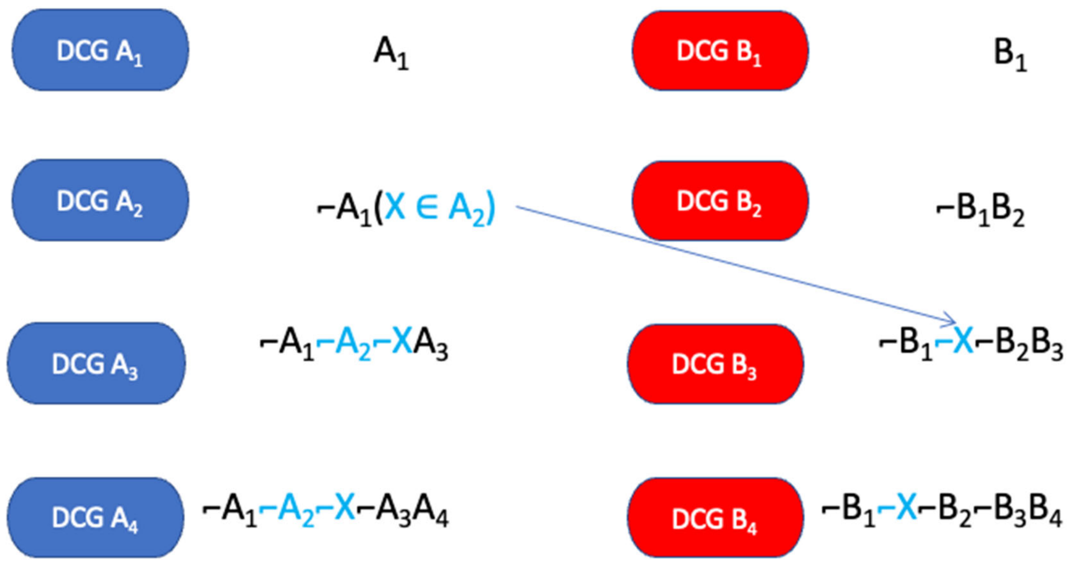

The algorithm iteratively seeks to place DXIs into DCGs. In this example, the earliest assignment of DXI_X occurs in HIER A (A_2). Thus, DXI_X will be reset to zero for that person in all subsequent DCG iterations. For any person with a DXI assigned to A_2, all of the remaining DXIs in HIER A and B are also reset to zero. The coefficient reflecting DXI_X is that of DCG A_2 without regard to lower ranked DCGs in HIER B, Other people with nonzero DXIs can still be assigned in subsequent iterations to lower ranked DCGs in both A and B hierarchies.

Notes: DXI is a Diagnostic Item and DCG is a Diagnostic Cost Group. A_i are sets of DXIs. ⌐ means not. DXI is a Diagnostic Item, and DCG is a Diagnostic Cost Group.

© 2024 Andriola C et al. *JAMA Health Forum*.

eFigure 1. Additive DXI Model Circulatory Coefficients Organized into 13 Hierarchies

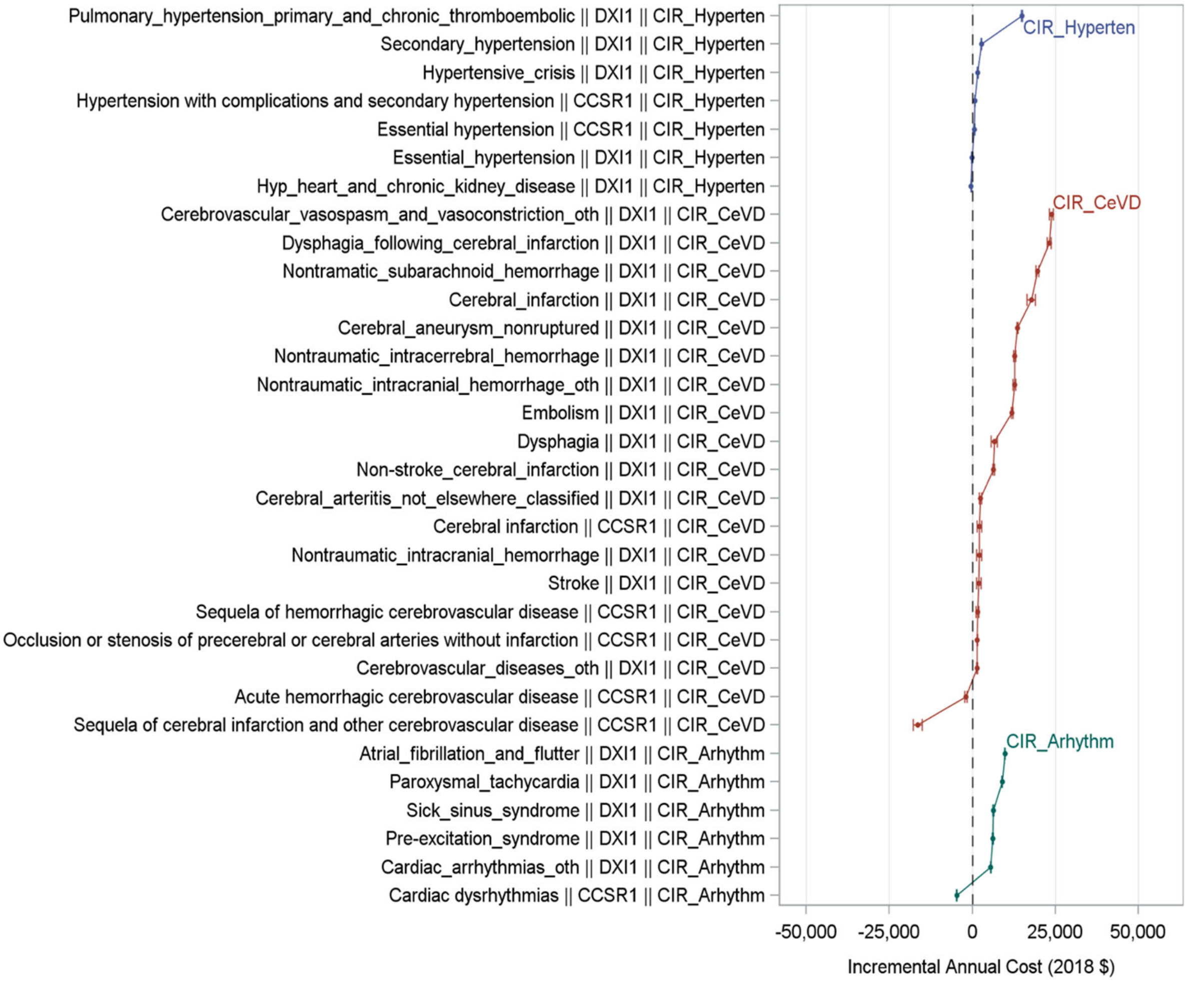

© 2024 Andriola C et al. *JAMA Health Forum*.

eFigure 1 (continued): Additive DXI Model Circulatory (CIR) Coefficients Organized into 13 Hierarchies

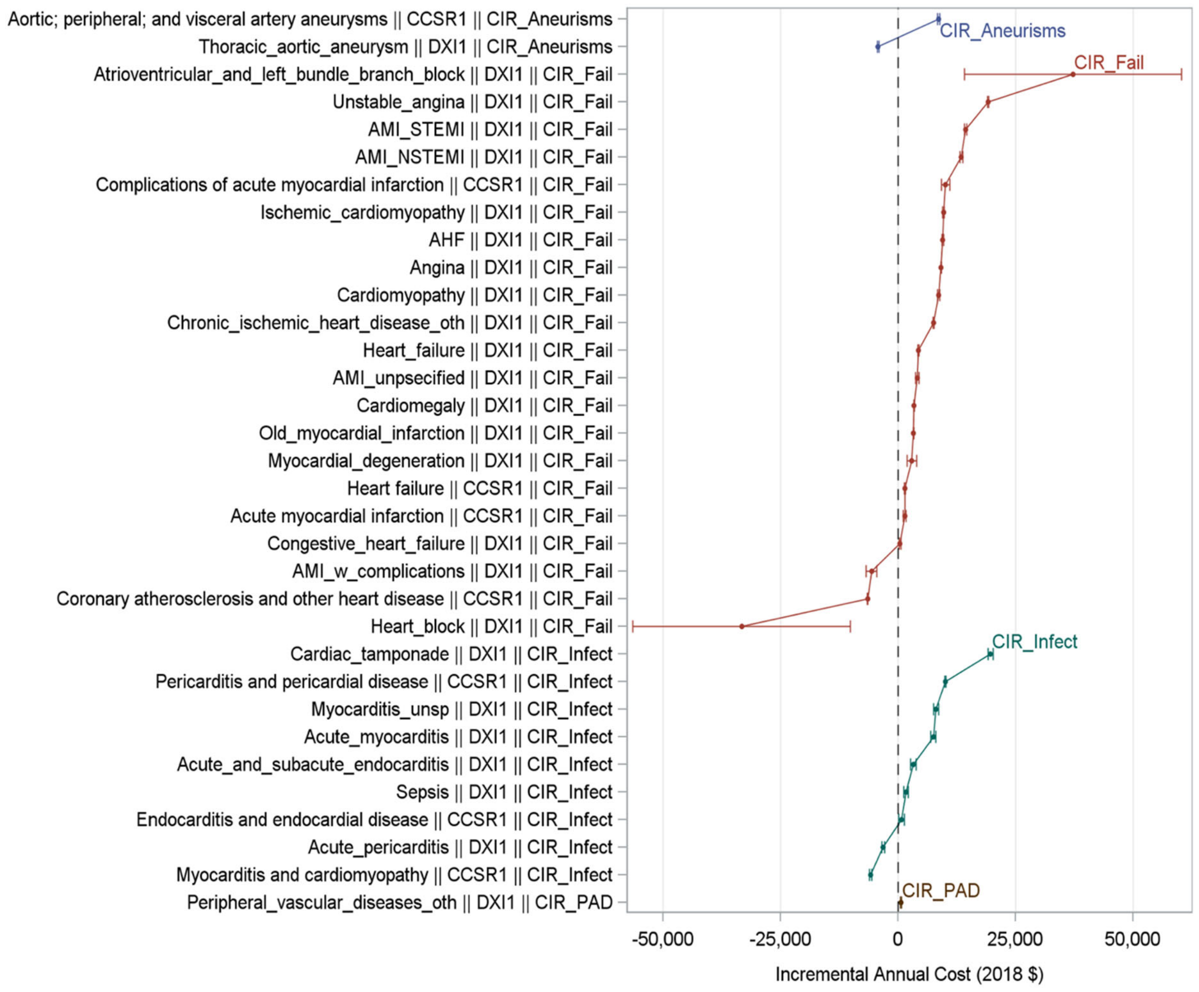

© 2024 Andriola C et al. *JAMA Health Forum*.

e**Figure 1 (continued)**: Additive DXI Model Circulatory (CIR) Coefficients Organized into 13 Hierarchies

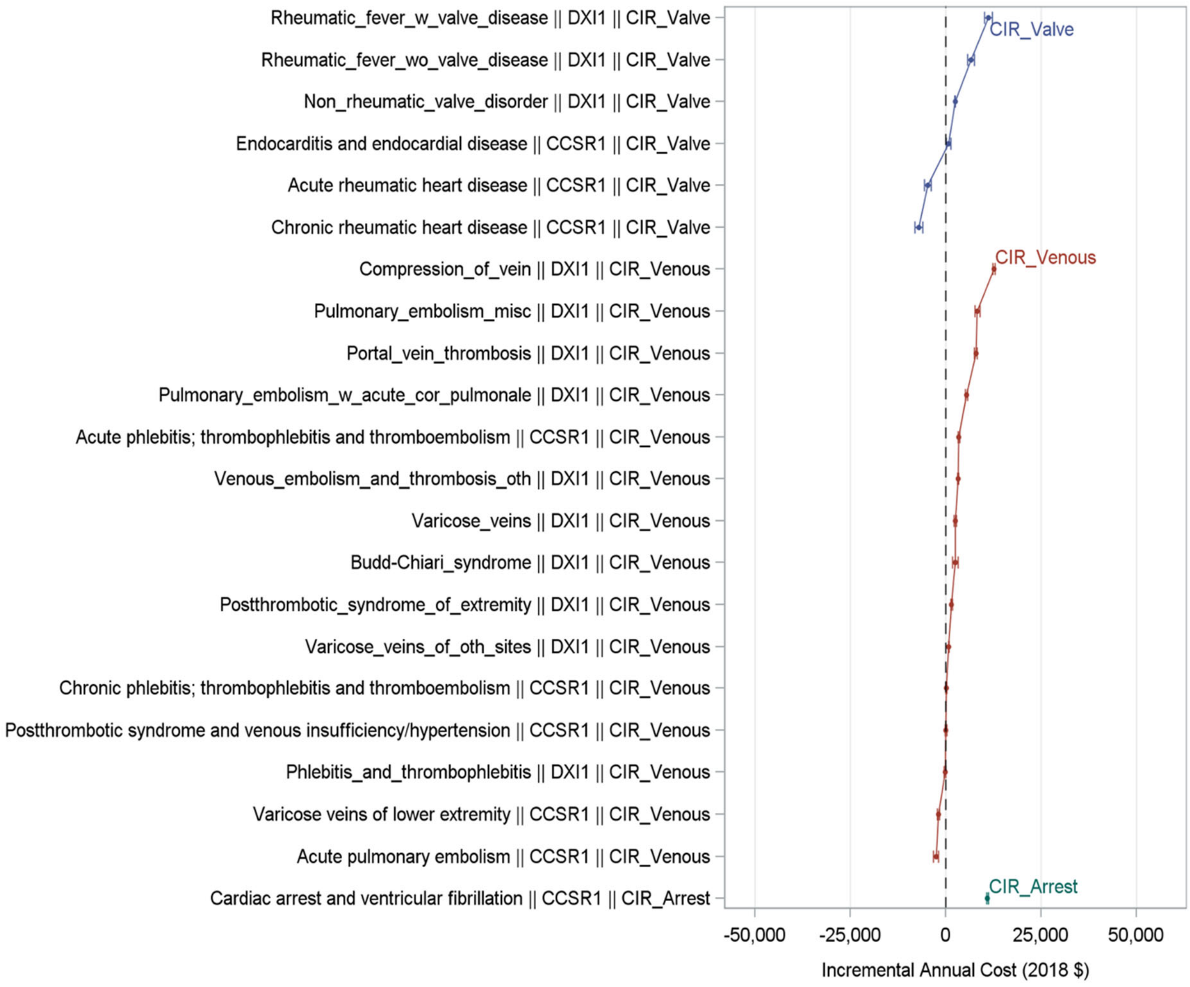

© 2024 Andriola C et al. *JAMA Health Forum*.

e**Figure 1 (continued)**: Additive DXI Model Circulatory (CIR) Coefficients Organized into 13 Hierarchies

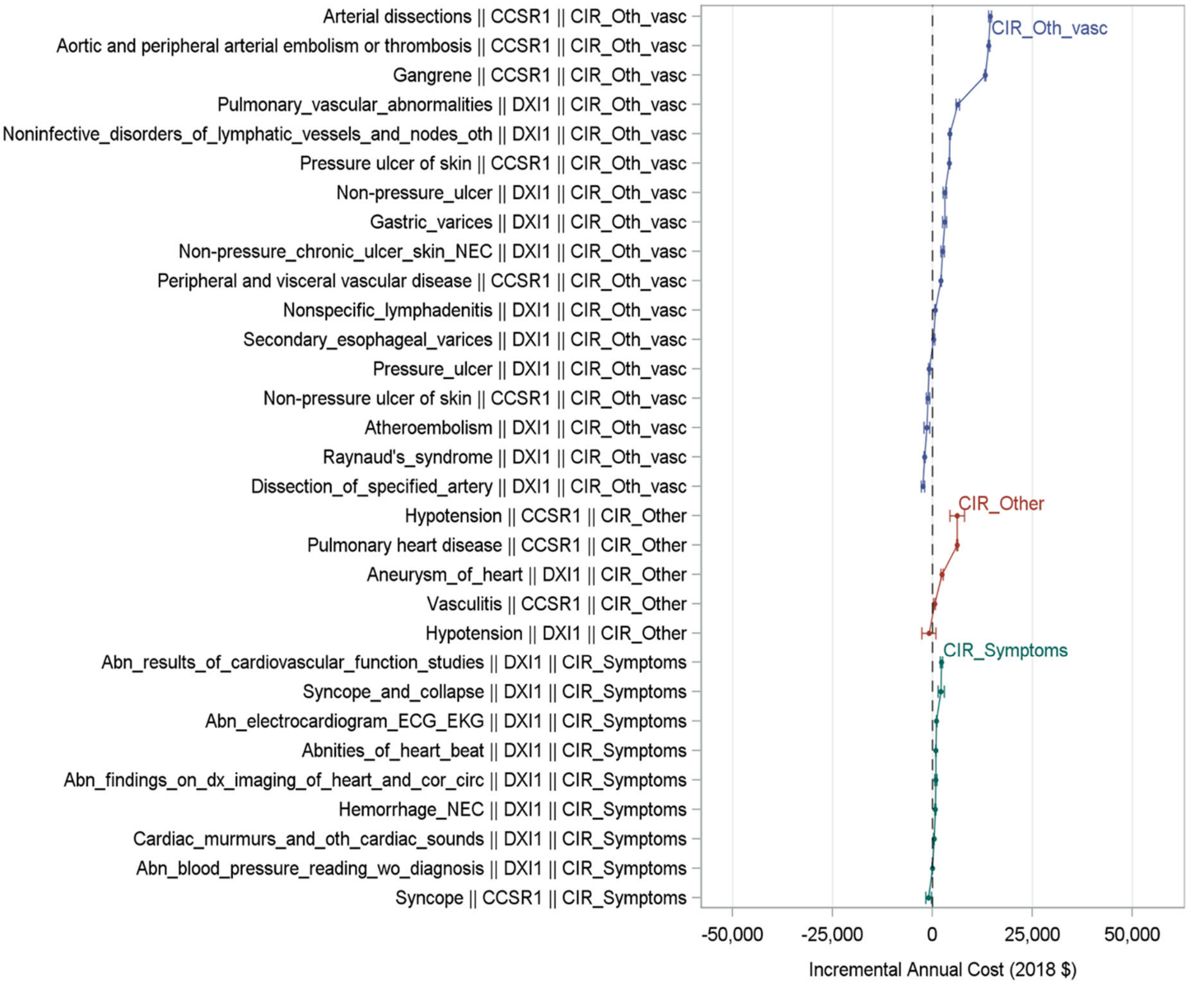

Notes: WLS is weighted least squares, CCSR is the Clinical Classifications Software Refined model, DXI is the Diagnostic Items model, and CIR is the Circulatory chapter. Plot whiskers correspond to 95% confidence intervals. Chapter assignment is based on the hierarchy mappings.

© 2024 Andriola C et al. *JAMA Health Forum*.

eFigure 2. DCG Model Coefficients for Sets of Circulatory DXIs, DCG Version 1.1

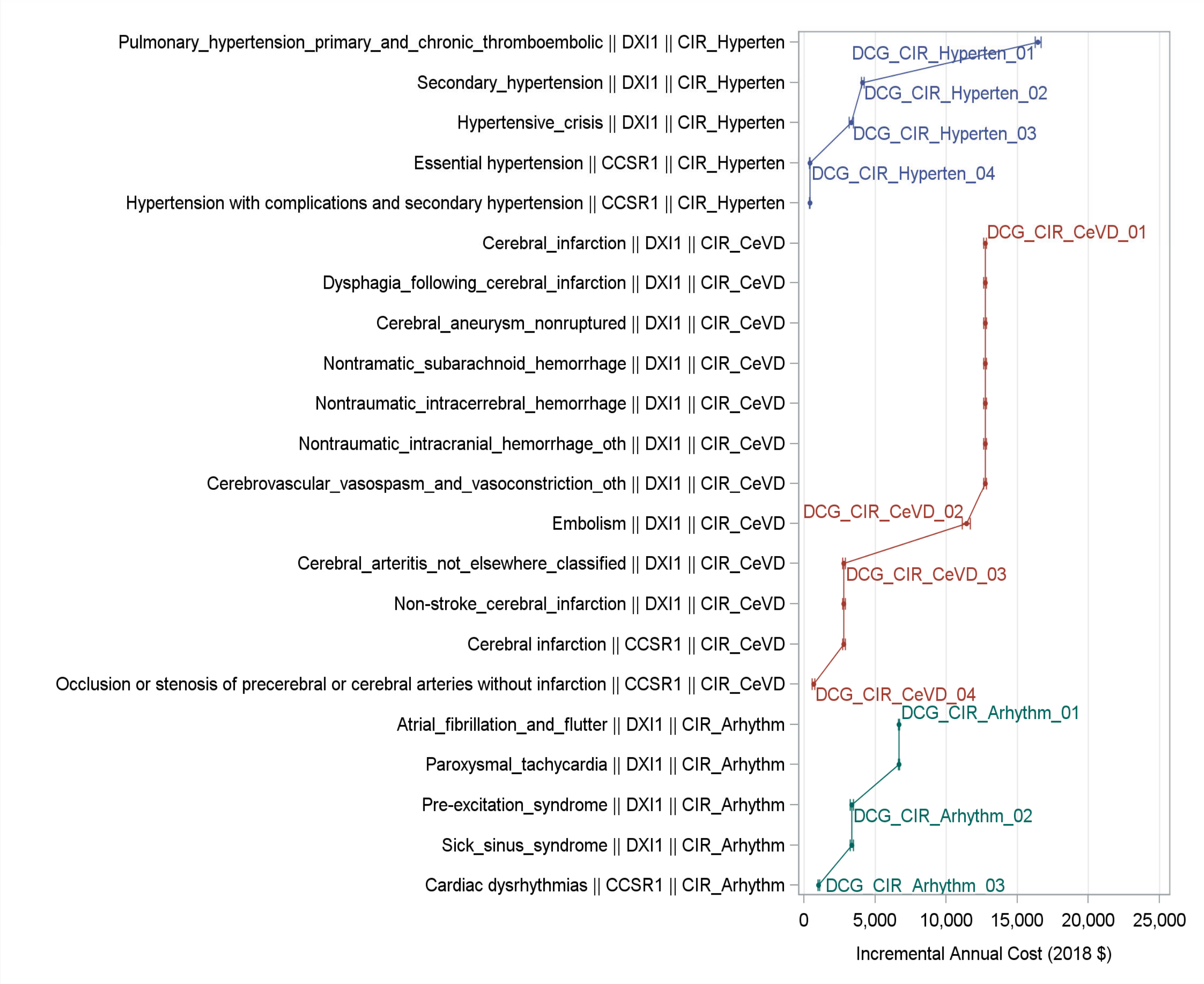

© 2024 Andriola C et al. *JAMA Health Forum*.

eFigure 2 (continued): DCG Model Coefficients for Sets of Circulatory (CIR) DXIs, DCG Version 1.1

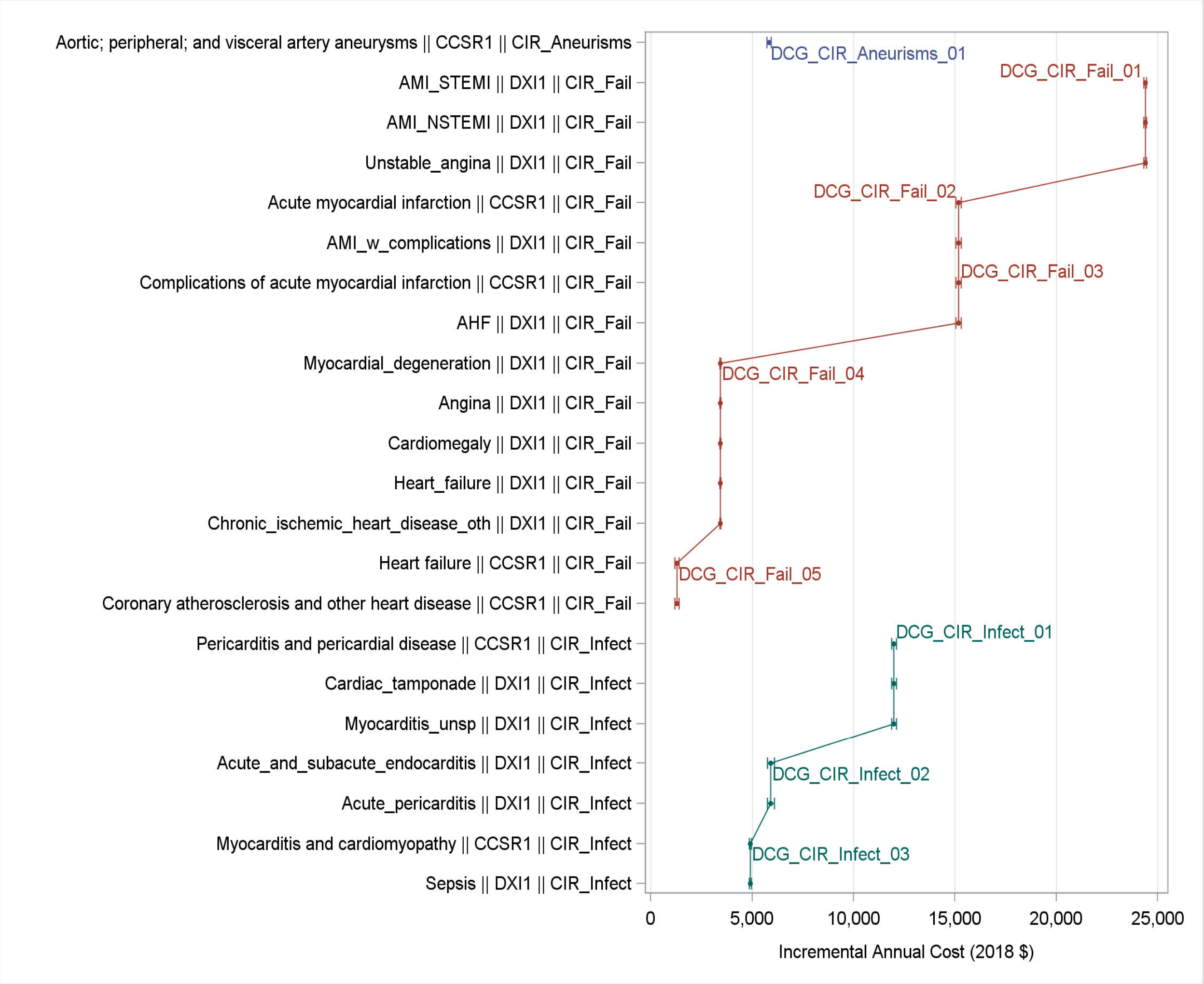

© 2024 Andriola C et al. *JAMA Health Forum*.

eFigure 2 (continued): DCG Model Coefficients for Sets of Circulatory (CIR) DXIs, DCG Version 1.1

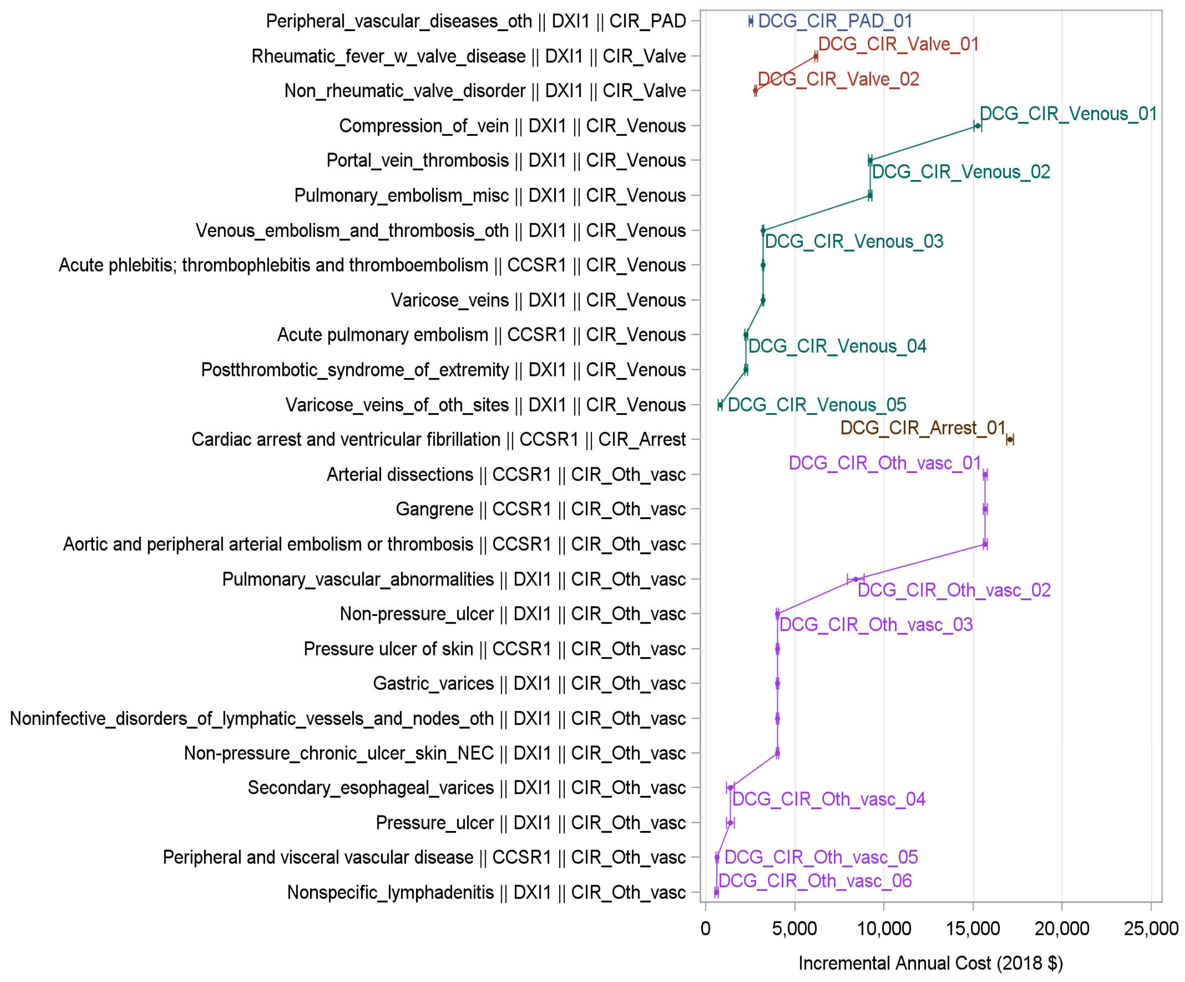

© 2024 Andriola C et al. *JAMA Health Forum*.

eFigure 2 (continued): DCG Model Coefficients for Sets of Circulatory (CIR) DXIs, DCG Version 1.1

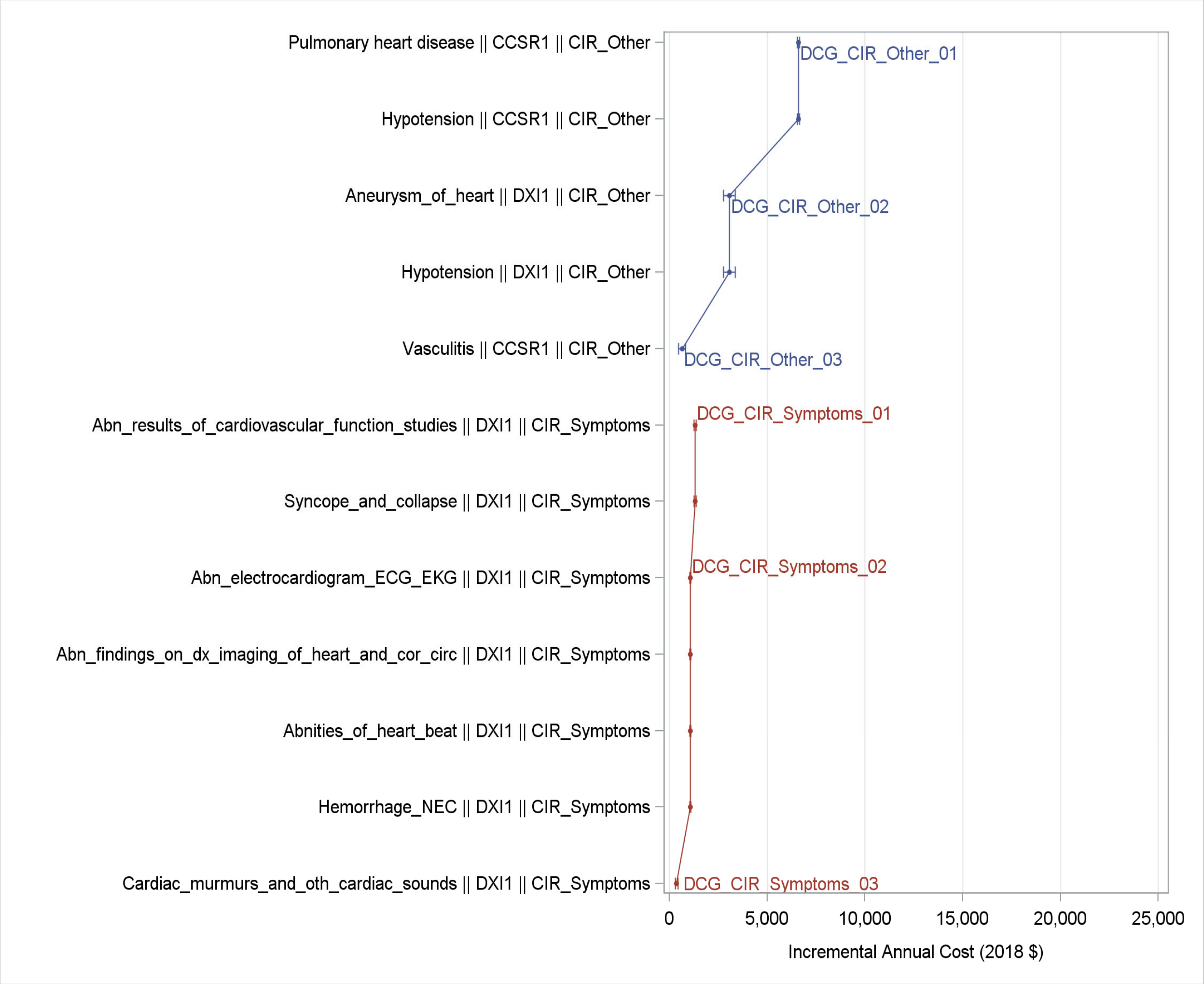

Notes: WLS is weighted least squares, CCSR is the Clinical Classifications Software Refined model, DXI is the Diagnostic Items model, DCG is the Diagnostic Cost Group, and CIR is the Circulatory chapter. Plot whiskers correspond to 95% confidence intervals. Chapter assignment is based on the hierarchy mappings.

© 2024 Andriola C et al. *JAMA Health Forum*.

eFigure 3. DCG Model Coefficients for Sets of Endocrine, Nutritional, and Metabolic DXIs, DCG Version 1.1

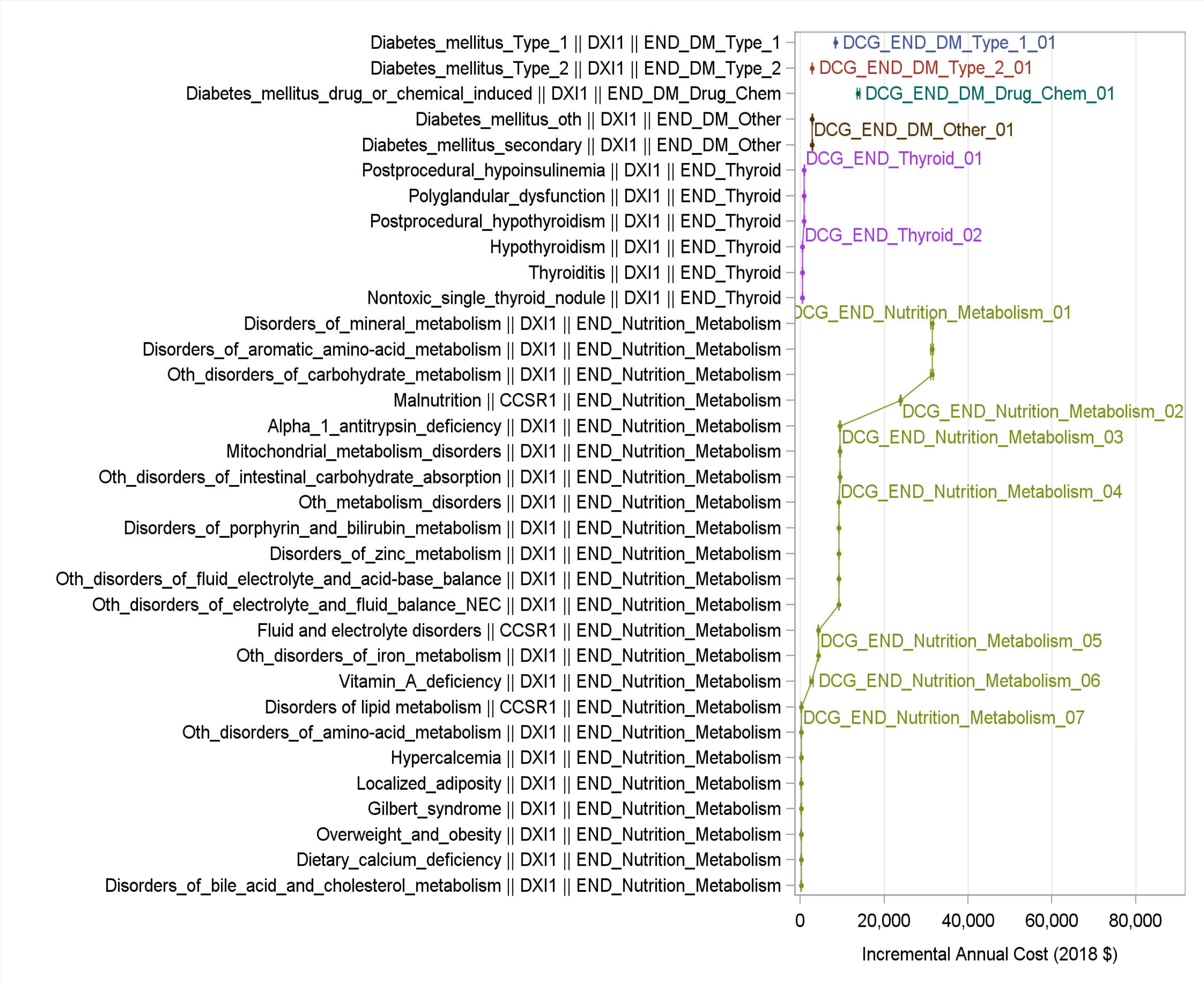

© 2024 Andriola C et al. *JAMA Health Forum*.

eFigure 3 (continued): DCG Model Coefficients for Sets of Endocrine, Nutritional, and Metabolic (END) DXIs, DCG version 1.1

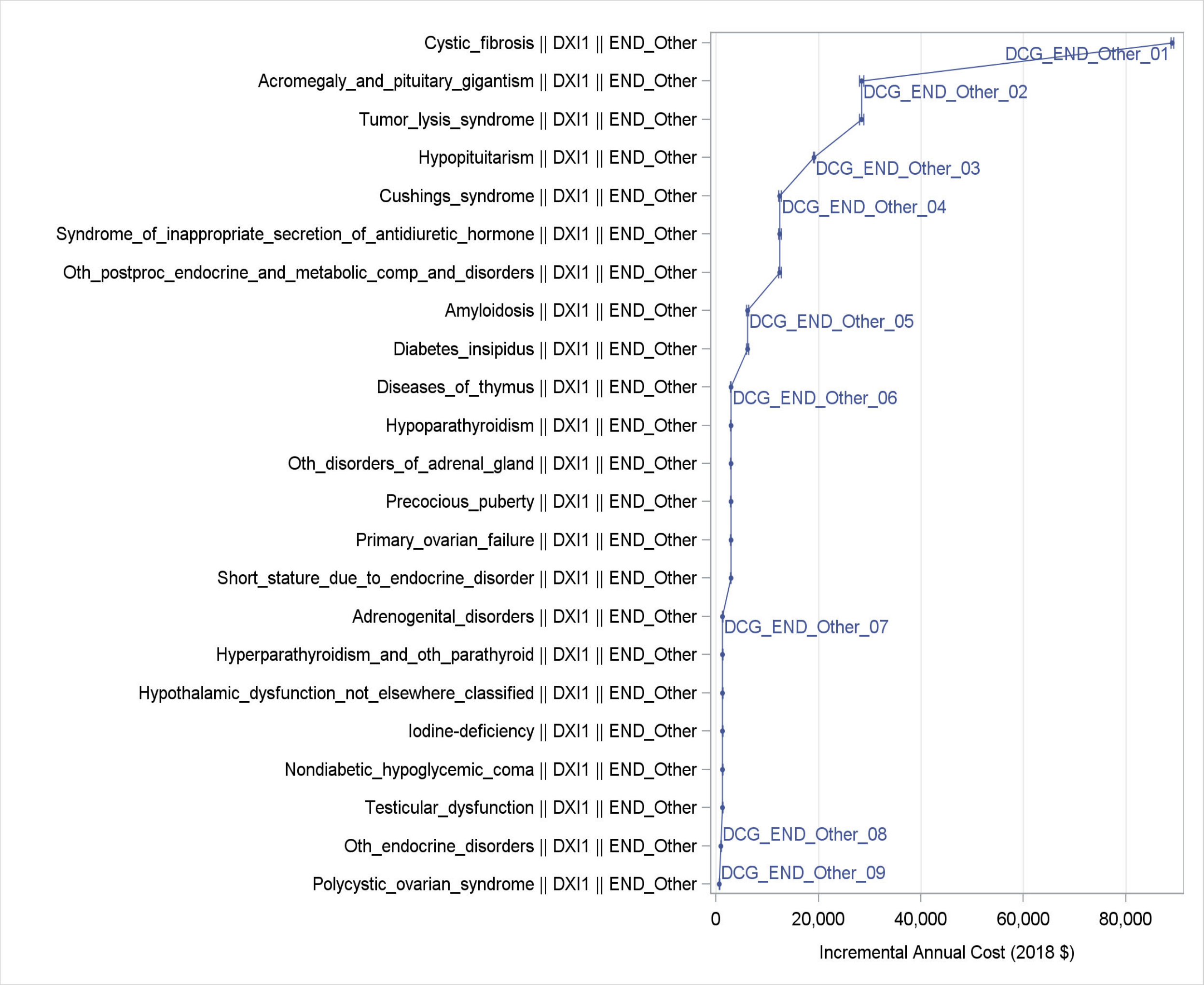

Notes: WLS is weighted least squares; CCSR is the Clinical Classifications Software Refined model; DXI is the Diagnostic Items model; DCG is the Diagnostic Cost Group; and END is the Endocrine, nutritional, and metabolic chapter. Plot whiskers correspond to 95% confidence intervals. Chapter assignment is based on the hierarchy mappings.

© 2024 Andriola C et al. *JAMA Health Forum*.

eFigure 4. DCG Model Coefficients for Sets of INJ DXIs, DCG Version 1.1

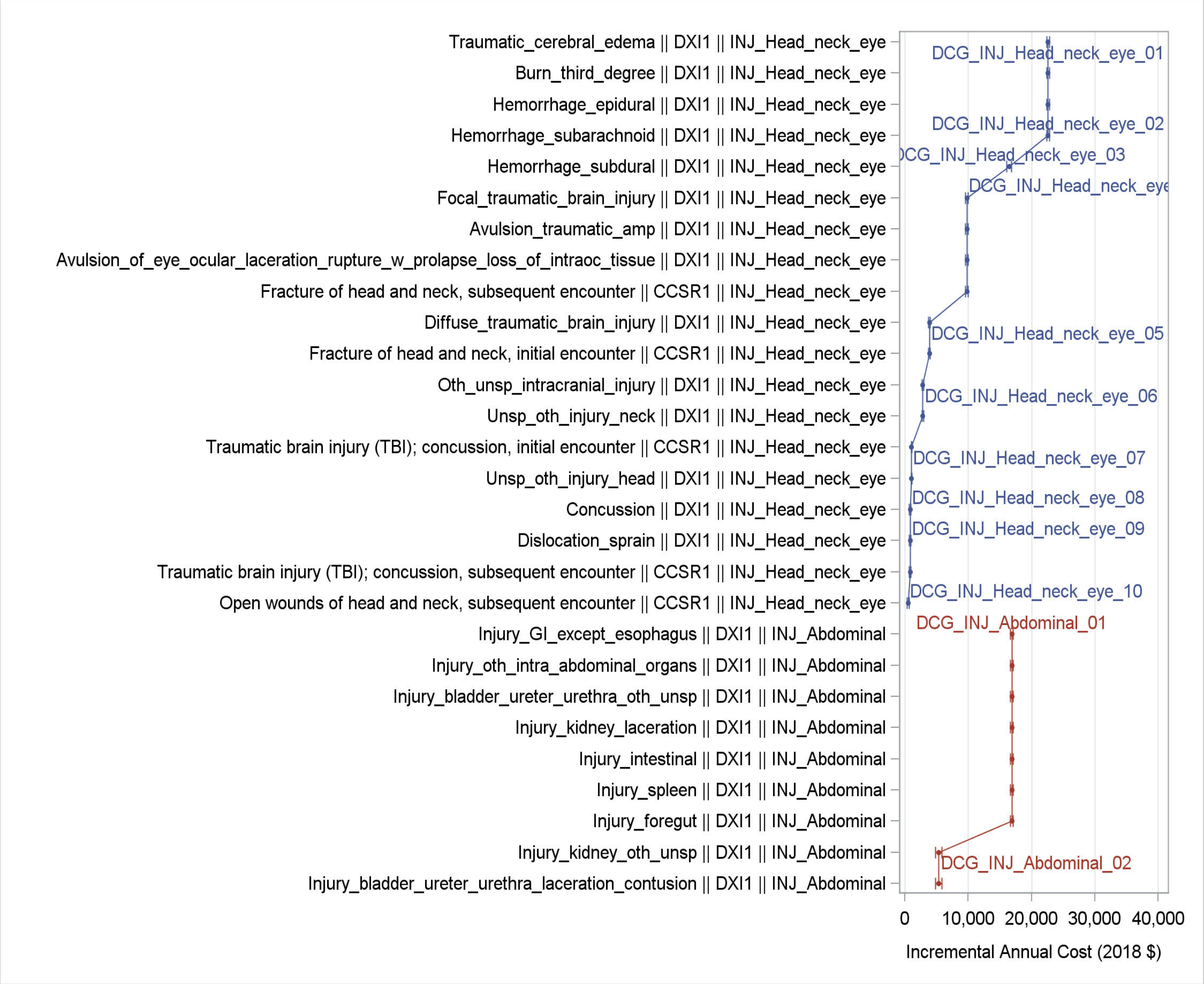

© 2024 Andriola C et al. *JAMA Health Forum*.

eFigure 4 (continued): DCG Model Coefficients for Sets of INJ DXIs, DCG version 1.1

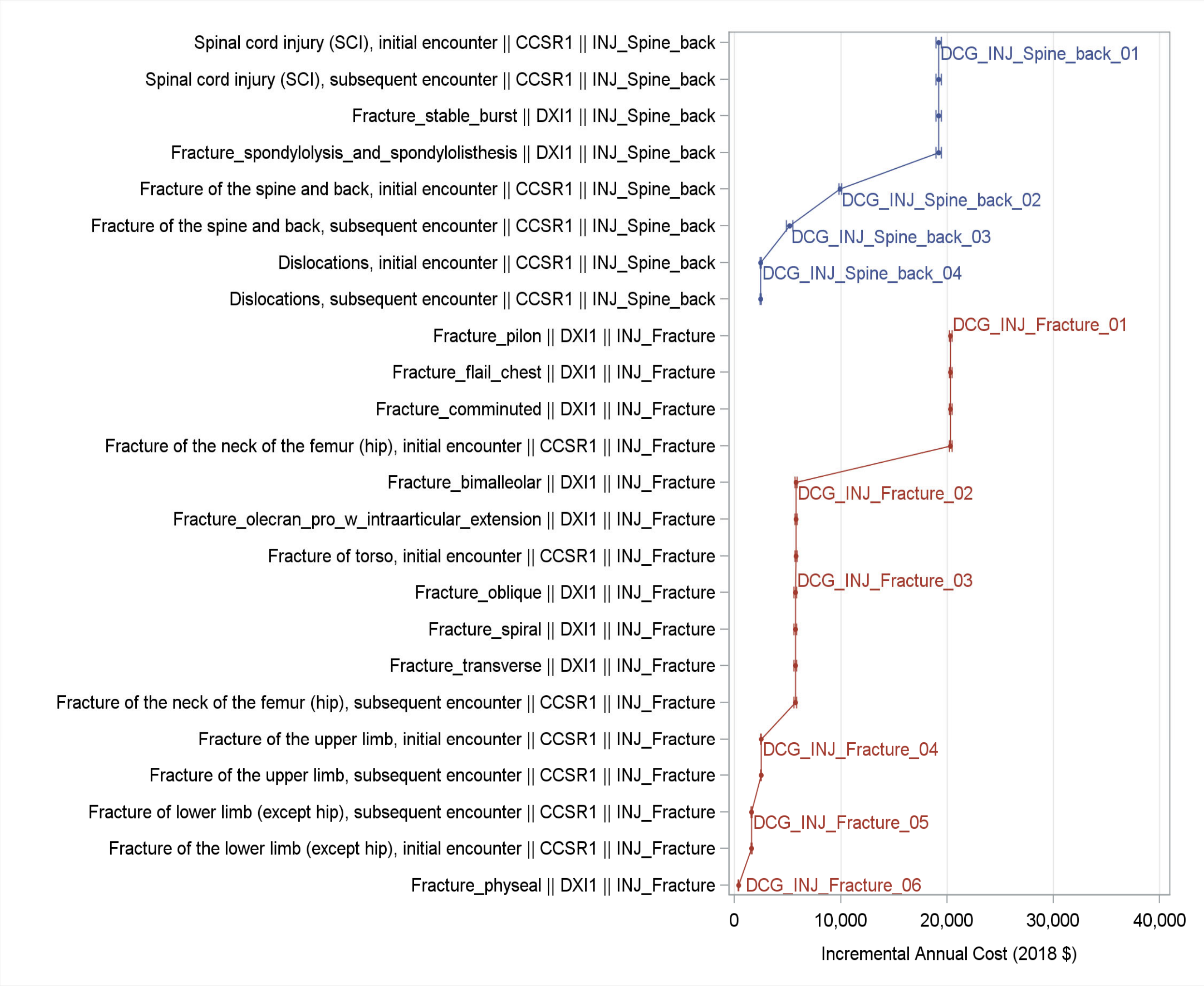

© 2024 Andriola C et al. *JAMA Health Forum*.

eFigure 4 (continued): DCG Model Coefficients for Sets of INJ DXIs, DCG version 1.1

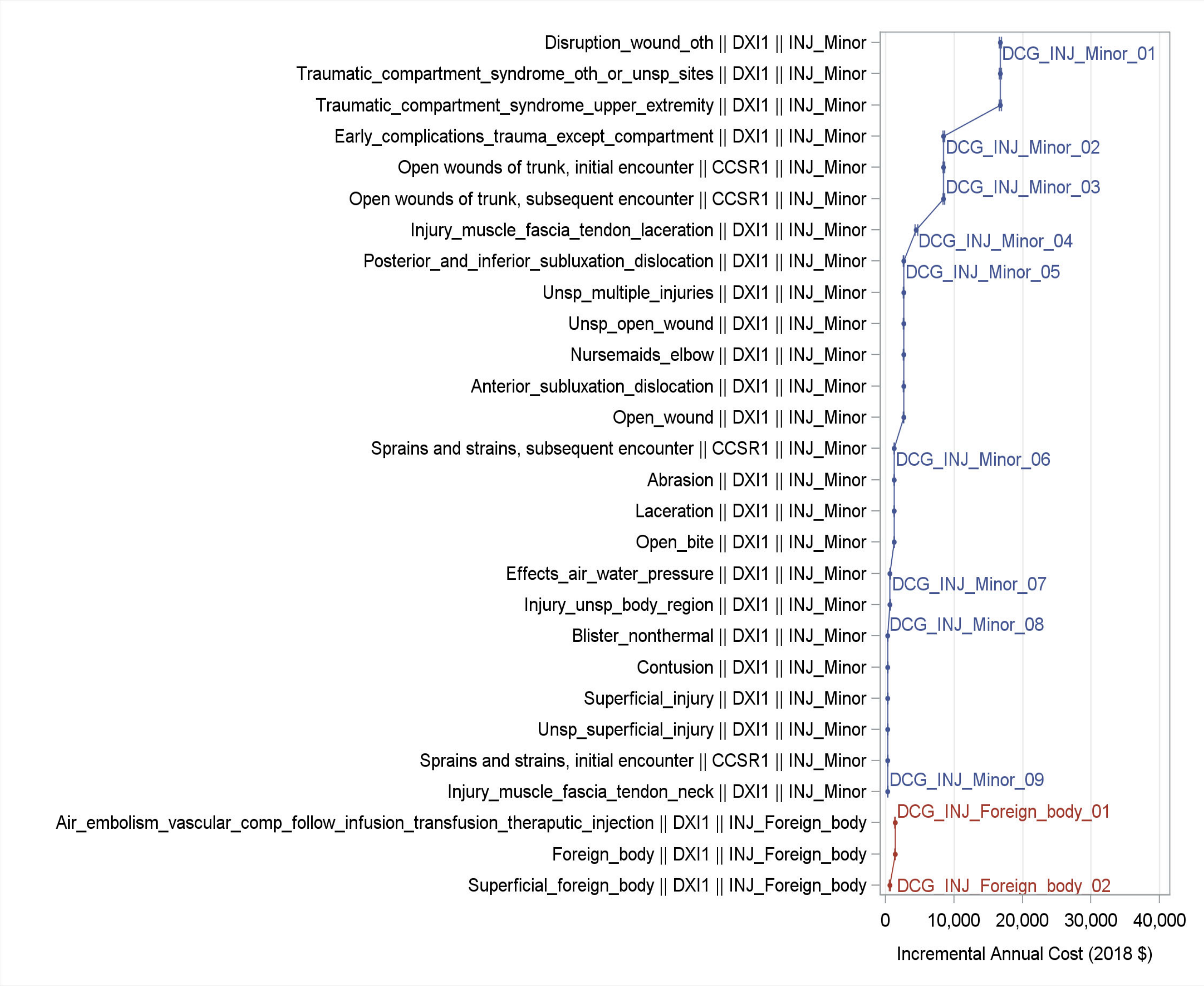

© 2024 Andriola C et al. *JAMA Health Forum*.

eFigure 4 (continued): DCG Model Coefficients for Sets of INJ DXIs, DCG version 1.1

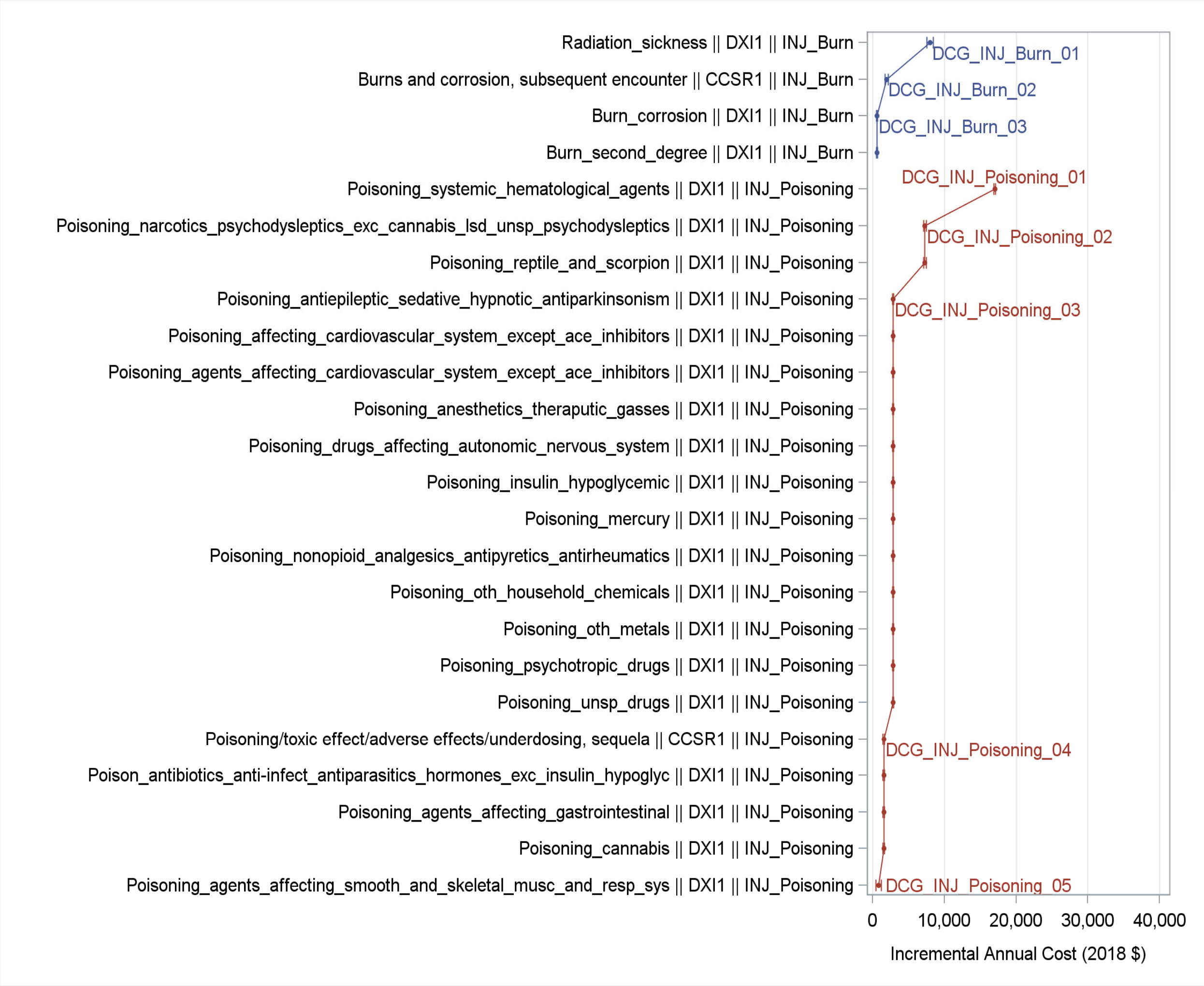

© 2024 Andriola C et al. *JAMA Health Forum*.

eFigure 4 (continued): DCG Model Coefficients for Sets of INJ DXIs, DCG version 1.1

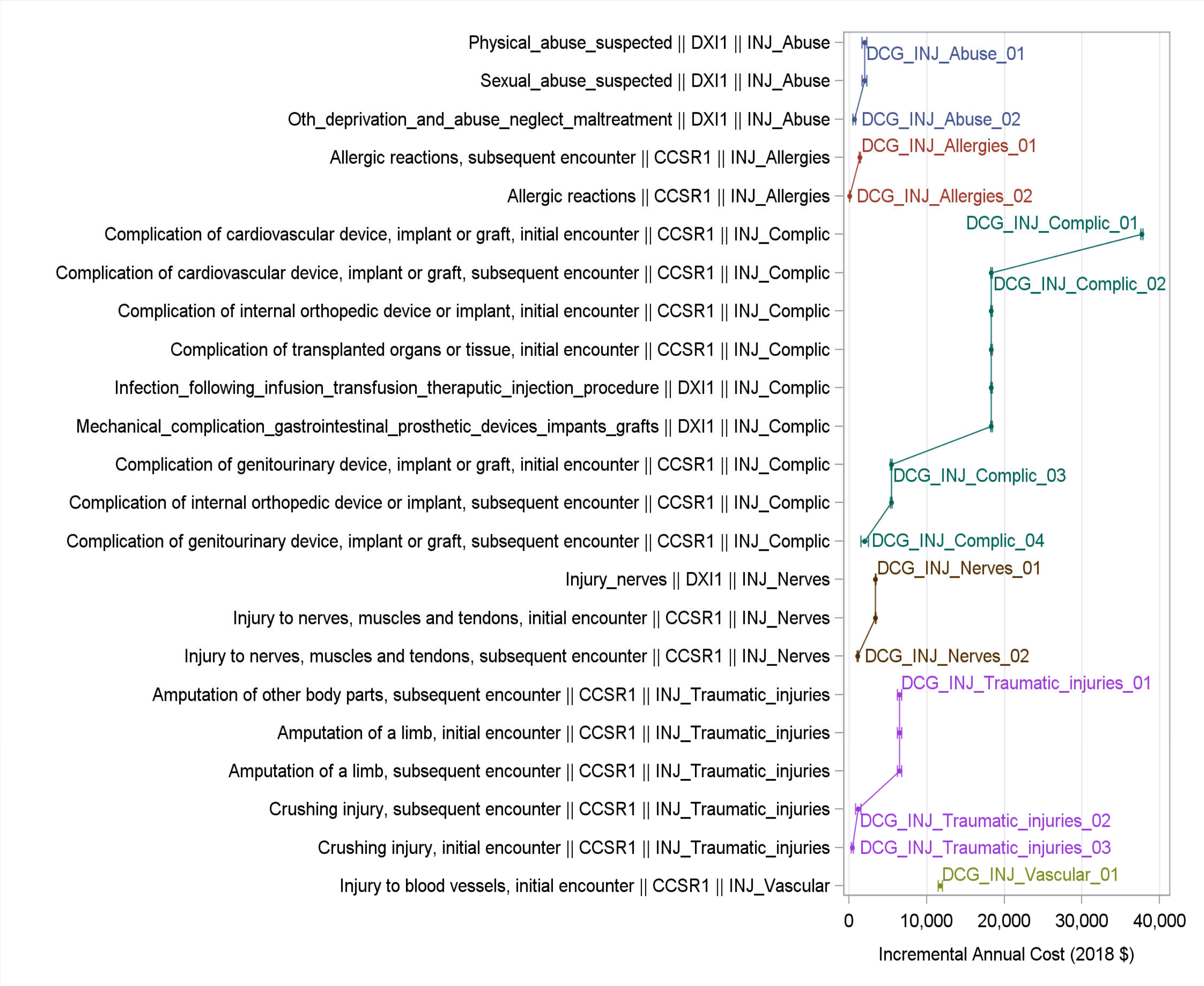

Notes: WLS is weighted least squares; CCSR is the Clinical Classifications Software Refined model; DXI is the Diagnostic Items model; DCG is the Diagnostic Cost Group; and INJ is the Injury, poisoning, and other external causes chapter. Plot whiskers correspond to 95% confidence intervals. Chapter assignment is based on the hierarchy mappings.

© 2024 Andriola C et al. *JAMA Health Forum*.

eTable 1. Modeling Principles Used in This Project as Expanded From Ash et al (2000)[6] in CMS-CIOO (2021)[7]

Principle 1: Diagnostic categories should be clinically meaningful.
Principle 2: Diagnostic categories should predict medical (including drug) expenditures.
Principle 3: Diagnostic categories that will affect payments should have adequate sample sizes to permit accurate and stable estimates of expenditures.
Principle 4: In creating an individual's clinical profile, hierarchies should be used to characterize the person's illness level within each disease process, while the effects of unrelated disease processes accumulate.
Principle 5: The diagnostic classification should encourage specific coding.
Principle 6: The diagnostic classification should not reward coding proliferation.
Principle 7: Providers should not be penalized for recording additional diagnoses (monotonicity).
Principle 8: The classification system should be internally consistent (transitive).
Principle 9: The diagnostic classification should assign all ICD-10-CM codes (exhaustive classification).
Principle 10: Discretionary diagnostic categories should be excluded from payment models.

Two new principles were added in this project:
Principle 11: Models should do well even on sets of rare diagnoses
Principle 12: Parsimonious models with fewer parameters are preferred.

© 2024 Andriola C et al. *JAMA Health Forum*.

eTable 2. Scale Used by Clinicians to Assign Appropriateness to Include Scores

We instructed the clinical review panels to use the following definitions for assigning ATI scores to each DXI.

0 => No concerns about using for payment
1 => Trivial concerns …
2 => Minor concerns …
3 => Meaningful concerns …
4 => Serious concerns …
5 => Major concerns: avoid using for payment

Panelists were informed that 4 might be a plausible threshold for exclusion from the model.

© 2024 Andriola C et al. *JAMA Health Forum*.

eTable 3. Summary Statistics From Development and Validation Samples

	Development Sample		Validation Sample	
	(N = 59,297,201)		(N = 6,604,259)	
Study variables	Mean	Std Dev	Mean	Std Dev
**Demographic:**				
Age	33.7	17.29	33.69	17.29
Female	51.48%		51.49%	
Months eligible	11.37	1.74	11.37	1.74
**Spending measures:**				
Total healthcare	$6,146	26,336	$6,167	26,405
Total healthcare topcoded at $250,000	$5,847	18,156	$5,862	18,159

© 2024 Andriola C et al. *JAMA Health Forum*.

eTable 4. Sensitivity Analysis: Validation Sample Measures of Alternative Model Tuning Parameters

	R-Square	Mean absolute error	Number of parameters	Rare disease mean error: mean residual of enrollee-years with any diagnosis rarer than 100 per million
**DCG: Base model with monotonicity restrictions**	**0.535**	**$4,114**	**624**	**-$73**
DCG: Base model without monotonicity	0.535	$4,113	661	-$71
**Simulation Parameters**				
Minimum sample sizes threshold				
DCG: N>500	0.535	$4,107	712	-$66
DCG: N>1000	0.535	$4,111	693	-$70
DCG: N>2000 (Base model without monotonicity)	0.535	$4,113	661	-$71
DCG: N>5000	0.534	$4,110	610	-$65
Maximum % difference threshold				
DCG: 10%	0.537	$4,095	934	-$62
DCG: 20%	0.536	$4,106	766	-$76
DCG: 30% (Base model without monotonicity)	0.535	$4,113	661	-$71
DCG: 40%	0.533	$4,121	584	-$63
Statistical significance threshold				
DCG: 0.01	0.534	$4,115	666	-$76
DCG: 0.001 (Base model without monotonicity)	0.535	$4,113	661	-$71
DCG: 0.0001	0.535	$4,113	655	-$69

Notes: DCG is the Diagnostic Cost Group algorithm.

© 2024 Andriola C et al. *JAMA Health Forum*.

eTable 5. Coefficients From DCG WLS Regression Best Incentives DCG ML Model

Dependent Variable is Total Health Spending Topcoded at 250 K (Parameters and regression results)

	Development Sample	DCG WLS Total Healthcare Spending Top-Coded at 250 K<br>K = 624<br>R-square in Validation Sample = 53.45%	
**Independent Variable Label**	**N**	**Coefficient**	**95% Confidence Interval**
DCG_AGE_Female_01	541,793	$711	($676, $745)
DCG_AGE_Female_02	8,184,277	$355	($346, $365)
DCG_AGE_Female_03	8,087,462	$194	($185, $203)
DCG_AGE_Female_04	2,459,762	$50	($34, $66)
DCG_AGE_Male_01	568,209	$751	($717, $786)
DCG_AGE_Male_02	10,294,636	$173	($165, $181)
DCG_AGE_Male_03	10,343,657	$62	($54, $70)
DCG_AGE_Male_04	1,910,660	$30	($13, $48)
DCG_BLD_Nutrit_anem_01	680,459	$951	($921, $981)
DCG_BLD_Hemo_anem_01	7,214	$22,588	($22,309, $22,866)
DCG_BLD_Hemo_anem_02	3,713	$12,568	($12,181, $12,955)
DCG_BLD_Hemo_anem_03	10,698	$10,582	($10,354, $10,810)
DCG_BLD_Hemo_anem_04	45,605	$677	($567, $788)
DCG_BLD_Aplastic_anem_01	14,163	$35,537	($35,323, $35,752)
DCG_BLD_Aplastic_anem_02	25,709	$23,730	($23,576, $23,885)
DCG_BLD_Aplastic_anem_03	65,878	$12,977	($12,870, $13,085)
DCG_BLD_Aplastic_anem_04	650,686	$3,587	($3,556, $3,618)
DCG_BLD_Coagulation_def_01	4,789	$79,492	($79,151, $79,833)
DCG_BLD_Coagulation_def_02	2,287	$23,927	($23,428, $24,426)
DCG_BLD_Coagulation_def_03	71,642	$10,202	($10,111, $10,293)
DCG_BLD_Coagulation_def_04	175,308	$4,781	($4,723, $4,839)
DCG_BLD_Coagulation_def_05	49,919	$2,351	($2,245, $2,458)
DCG_BLD_Blood_form_org_01	21,877	$38,344	($38,167, $38,521)
DCG_BLD_Blood_form_org_02	4,058	$25,225	($24,852, $25,599)
DCG_BLD_Blood_form_org_03	44,358	$8,652	($8,532, $8,772)
DCG_BLD_Blood_form_org_04	8,971	$4,413	($4,156, $4,670)
DCG_BLD_Immune_mech_01	9,559	$42,168	($41,926, $42,409)
DCG_BLD_Immune_mech_02	144,647	$9,679	($9,615, $9,742)
DCG_BLD_Other_01	433,215	$4,531	($4,491, $4,572)
DCG_BLD_Other_02	74,136	$635	($545, $726)
DCG_BLD_Intraop_postproc_01	110,362	$27,178	($27,103, $27,252)
DCG_BLD_Symptoms_01	3,108	$18,231	($17,808, $18,655)
DCG_BLD_Symptoms_02	10,633	$4,187	($3,958, $4,416)
DCG_BLD_Symptoms_03	6,092	$1,660	($1,358, $1,962)
DCG_BLD_Symptoms_04	268,870	$1,059	($1,012, $1,105)
DCG_CIR_Hyperten_01	14,617	$16,452	($16,250, $16,655)

© 2024 Andriola C et al. *JAMA Health Forum*.

	Development Sample	DCG WLS Total Healthcare Spending Top-Coded at 250 K<br>K = 624<br>R-square in Validation Sample = 53.45%	
**Independent Variable Label**	**N**	**Coefficient**	**95% Confidence Interval**
DCG_CIR_Hyperten_02	53,198	$4,109	($4,006, $4,212)
DCG_CIR_Hyperten_03	29,542	$3,307	($3,169, $3,446)
DCG_CIR_Hyperten_04	7,098,674	$392	($380, $403)
DCG_CIR_CeVD_01	77,363	$12,724	($12,625, $12,823)
DCG_CIR_CeVD_02	6,515	$11,400	($11,106, $11,694)
DCG_CIR_CeVD_03	67,767	$2,795	($2,702, $2,889)
DCG_CIR_CeVD_04	79,398	$651	($566, $736)
DCG_CIR_Arhythm_01	425,055	$6,678	($6,640, $6,716)
DCG_CIR_Arhythm_02	51,329	$3,329	($3,219, $3,440)
DCG_CIR_Arhythm_03	216,088	$1,017	($966, $1,068)
DCG_CIR_Aneurisms_01	66,311	$5,824	($5,732, $5,917)
DCG_CIR_Fail_01	130,254	$24,388	($24,320, $24,456)
DCG_CIR_Fail_02	2,537	$15,184	($15,054, $15,314)
DCG_CIR_Fail_03	33,965	$15,184	($15,054, $15,314)
DCG_CIR_Fail_04	765,122	$3,430	($3,400, $3,459)
DCG_CIR_Fail_05	59,135	$1,286	($1,188, $1,384)
DCG_CIR_Infect_01	41,472	$11,989	($11,871, $12,106)
DCG_CIR_Infect_02	20,023	$5,909	($5,741, $6,077)
DCG_CIR_Infect_03	172,523	$4,895	($4,835, $4,956)
DCG_CIR_PAD_01	113,689	$2,521	($2,432, $2,609)
DCG_CIR_Valve_01	121,123	$6,169	($6,100, $6,239)
DCG_CIR_Valve_02	488,733	$2,773	($2,737, $2,809)
DCG_CIR_Venous_01	12,468	$15,245	($15,029, $15,461)
DCG_CIR_Venous_02	68,702	$9,213	($9,121, $9,305)
DCG_CIR_Venous_03	363,109	$3,193	($3,153, $3,233)
DCG_CIR_Venous_04	83,225	$2,242	($2,159, $2,324)
DCG_CIR_Venous_05	45,944	$785	($674, $896)
DCG_CIR_Arrest_01	19,504	$17,051	($16,868, $17,234)
DCG_CIR_Oth_vasc_01	39,413	$15,670	($15,547, $15,794)
DCG_CIR_Oth_vasc_02	2,564	$8,400	($7,931, $8,869)
DCG_CIR_Oth_vasc_03	157,935	$3,998	($3,936, $4,060)
DCG_CIR_Oth_vasc_04	12,725	$1,353	($1,128, $1,578)
DCG_CIR_Oth_vasc_05	236,050	$623	($561, $684)
DCG_CIR_Oth_vasc_06	69,678	$582	($493, $672)
DCG_CIR_Other_01	219,107	$6,584	($6,529, $6,639)
DCG_CIR_Other_02	5,979	$3,054	($2,746, $3,362)
DCG_CIR_Other_03	30,885	$639	($466, $813)
DCG_CIR_Symptoms_01	239,144	$1,309	($1,261, $1,358)
DCG_CIR_Symptoms_02	601,496	$1,057	($1,026, $1,088)
DCG_CIR_Symptoms_03	126,119	$356	($289, $423)

© 2024 Andriola C et al. *JAMA Health Forum*.

Independent Variable Label	Development Sample N	DCG WLS Total Healthcare Spending Top-Coded at 250 K K = 624 R-square in Validation Sample = 53.45% Coefficient	95% Confidence Interval
DCG_DIG_Mouth_01	12,246	$15,487	($15,272, $15,703)
DCG_DIG_Mouth_02	2,192	$6,112	($5,608, $6,616)
DCG_DIG_Mouth_03	42,272	$1,348	($1,233, $1,463)
DCG_DIG_Mouth_04	132,673	$609	($544, $674)
DCG_DIG_Esophagus_01	188,623	$2,873	($2,817, $2,930)
DCG_DIG_Esophagus_02	2,727,226	$1,263	($1,247, $1,279)
DCG_DIG_Stomach_01	8,792	$16,666	($16,413, $16,919)
DCG_DIG_Stomach_02	736,183	$1,384	($1,354, $1,415)
DCG_DIG_Ulcer_01	32,992	$14,002	($13,866, $14,137)
DCG_DIG_Ulcer_02	99,488	$1,008	($931, $1,085)
DCG_DIG_Intestine_01	118,320	$29,006	($28,936, $29,075)
DCG_DIG_Intestine_02	22,990	$17,929	($17,748, $18,110)
DCG_DIG_Intestine_03	7,333	$10,385	($10,103, $10,667)
DCG_DIG_Intestine_04	31,863	$3,577	($3,443, $3,711)
DCG_DIG_Intestine_05	651,676	$594	($562, $626)
DCG_DIG_Colon_01	101,683	$14,805	($14,730, $14,880)
DCG_DIG_Colon_02	129,697	$9,687	($9,618, $9,755)
DCG_DIG_Colon_03	8,424	$1,718	($1,460, $1,975)
DCG_DIG_Colon_04	632,106	$959	($929, $990)
DCG_DIG_Colon_05	363,756	$425	($386, $465)
DCG_DIG_Colon_06	141,329	$325	($248, $403)
DCG_DIG_Constipation_01	1,050,245	$1,776	($1,752, $1,800)
DCG_DIG_Functional_Diarrhea_01	5,387	$12,779	($12,438, $13,121)
DCG_DIG_Appendix_01	75,122	$13,710	($13,623, $13,796)
DCG_DIG_Appendix_02	46,897	$2,533	($2,409, $2,657)
DCG_DIG_Anus_01	17,388	$8,075	($7,895, $8,255)
DCG_DIG_Anus_02	947,851	$967	($942, $991)
DCG_DIG_Anus_03	146,195	$967	($942, $991)
DCG_DIG_Anus_04	94,506	$967	($942, $991)
DCG_DIG_Anus_05	150,160	$645	($583, $708)
DCG_DIG_Liver_01	13,607	$20,012	($19,798, $20,226)
DCG_DIG_Liver_02	15,112	$6,393	($6,200, $6,586)
DCG_DIG_Liver_03	37,237	$3,806	($3,676, $3,935)
DCG_DIG_Liver_04	37,471	$1,785	($1,663, $1,907)
DCG_DIG_Biliary_01	251,944	$9,132	($9,084, $9,180)
DCG_DIG_Biliary_02	2,991	$967	($536, $1,398)
DCG_DIG_Pancreas_01	3,457	$17,455	($17,052, $17,857)
DCG_DIG_Pancreas_02	50,937	$8,683	($8,576, $8,789)
DCG_DIG_Pancreas_03	108,160	$1,310	($1,238, $1,382)
DCG_DIG_Hernia_Abdominal_01	331,025	$3,606	($3,563, $3,649)

© 2024 Andriola C et al. *JAMA Health Forum*.

	Development Sample	DCG WLS Total Healthcare Spending Top-Coded at 250 K<br>K = 624<br>R-square in Validation Sample = 53.45%	
**Independent Variable Label**	**N**	**Coefficient**	**95% Confidence Interval**
DCG_DIG_Hernia_Abdominal_02	263,343	$2,637	($2,588, $2,686)
DCG_DIG_Visceral_Vasc_Dis_01	3,915	$7,949	($7,568, $8,329)
DCG_DIG_Visceral_Vasc_Dis_02	4,377	$2,869	($2,508, $3,231)
DCG_DIG_Other_01	52,296	$14,203	($14,097, $14,309)
DCG_DIG_Other_02	264,812	$450	($392, $508)
DCG_DIG_Intraop_postproc_01	60,799	$7,778	($7,680, $7,875)
DCG_DIG_Symptoms_01	4,484	$12,875	($12,521, $13,228)
DCG_DIG_Symptoms_02	1,220,199	$1,520	($1,498, $1,542)
DCG_DIG_Symptoms_03	17,920	$1,520	($1,498, $1,542)
DCG_DIG_Symptoms_04	8,413	$1,470	($1,213, $1,728)
DCG_DIG_Symptoms_05	1,849,381	$562	($544, $581)
DCG_DIG_Symptoms_06	61,594	$299	($204, $394)
DCG_EAR_Hearing_impairment_01	731,594	$1,217	($1,188, $1,246)
DCG_EAR_Hearing_impairment_02	63,397	$408	($319, $497)
DCG_EAR_Hearing_impairment_03	7,611	$408	($319, $497)
DCG_EAR_Infection_medically_managed_02	54,042	$308	($206, $410)
DCG_EAR_Probable_surgical_management_01	15,859	$4,920	($4,731, $5,110)
DCG_EAR_Probable_surgical_management_02	15,124	$2,594	($2,401, $2,786)
DCG_EAR_Probable_surgical_management_03	208,521	$1,156	($1,104, $1,209)
DCG_EAR_Probable_surgical_management_04	19,646	$590	($422, $759)
DCG_EAR_Probable_surgical_management_05	66,871	$222	($131, $314)
DCG_EAR_Intraop_postop_01	3,718	$1,718	($1,329, $2,107)
DCG_END_DM_Type_1_01	274,086	$8,379	($8,333, $8,426)
DCG_END_DM_Type_2_01	2,495,734	$2,802	($2,785, $2,820)
DCG_END_DM_Drug_Chem_01	5,395	$13,798	($13,475, $14,121)
DCG_END_DM_Other_01	118,530	$2,780	($2,709, $2,851)
DCG_END_Thyroid_01	184,542	$862	($805, $920)
DCG_END_Thyroid_02	2,386,541	$517	($500, $533)
DCG_END_Nutrition_Metabolism_01	4,790	$31,393	($31,052, $31,734)
DCG_END_Nutrition_Metabolism_02	55,366	$23,843	($23,737, $23,949)
DCG_END_Nutrition_Metabolism_03	52,347	$9,385	($9,281, $9,488)
DCG_END_Nutrition_Metabolism_04	282,559	$9,163	($9,115, $9,211)
DCG_END_Nutrition_Metabolism_05	567,375	$4,272	($4,240, $4,305)
DCG_END_Nutrition_Metabolism_06	3,033	$2,626	($2,198, $3,055)
DCG_END_Nutrition_Metabolism_07	8,366,756	$151	($140, $162)
DCG_END_Other_01	7,949	$89,085	($88,817, $89,353)
DCG_END_Other_02	2,983	$28,370	($27,937, $28,803)
DCG_END_Other_03	37,289	$19,110	($18,987, $19,233)
DCG_END_Other_04	11,138	$12,443	($12,218, $12,667)
DCG_END_Other_05	8,908	$6,136	($5,886, $6,386)

© 2024 Andriola C et al. *JAMA Health Forum*.

	Development Sample	DCG WLS Total Healthcare Spending Top-Coded at 250 K<br>K = 624<br>R-square in Validation Sample = 53.45%	
**Independent Variable Label**	**N**	**Coefficient**	**95% Confidence Interval**
DCG_END_Other_06	156,463	$2,863	($2,802, $2,923)
DCG_END_Other_07	744,706	$1,242	($1,214, $1,270)
DCG_END_Other_08	84,734	$936	($855, $1,018)
DCG_END_Other_09	211,730	$623	($571, $675)
DCG_EYE_Ocular_adnexa_orbit_01	2,705	$8,421	($7,966, $8,875)
DCG_EYE_Ocular_adnexa_orbit_02	29,470	$2,729	($2,591, $2,867)
DCG_EYE_Ocular_adnexa_orbit_03	112,551	$1,342	($1,271, $1,413)
DCG_EYE_Ocular_adnexa_orbit_04	194,091	$396	($342, $450)
DCG_EYE_Ocular_adnexa_orbit_05	587,892	$271	($239, $303)
DCG_EYE_Anterior_segment_01	80,321	$2,441	($2,357, $2,526)
DCG_EYE_Anterior_segment_02	32,322	$1,314	($1,214, $1,414)
DCG_EYE_Anterior_segment_03	21,416	$1,314	($1,214, $1,414)
DCG_EYE_Anterior_segment_04	2,006	$1,314	($1,214, $1,414)
DCG_EYE_Anterior_segment_05	1,536,995	$279	($259, $299)
DCG_EYE_Eyeball_lens_01	15,137	$3,379	($3,187, $3,572)
DCG_EYE_Eyeball_lens_02	1,124,882	$331	($306, $356)
DCG_EYE_Glaucoma_or_suspect_01	51,345	$3,072	($2,967, $3,177)
DCG_EYE_Glaucoma_or_suspect_02	3,913	$1,784	($1,407, $2,161)
DCG_EYE_Glaucoma_or_suspect_03	37,471	$986	($863, $1,109)
DCG_EYE_Glaucoma_or_suspect_04	463,720	$181	($145, $216)
DCG_EYE_Strabismus_ocular_motility_01	16,439	$3,624	($3,436, $3,813)
DCG_EYE_Strabismus_ocular_motility_02	136,825	$1,295	($1,230, $1,359)
DCG_EYE_Strabismus_ocular_motility_04	21,931	$535	($373, $698)
DCG_EYE_Neuro-ophthmalogic_01	6,207	$10,381	($10,079, $10,683)
DCG_EYE_Neuro-ophthmalogic_02	5,833	$6,637	($6,326, $6,948)
DCG_EYE_Neuro-ophthmalogic_03	56,094	$3,017	($2,914, $3,121)
DCG_EYE_Neuro-ophthmalogic_04	60,979	$1,802	($1,705, $1,898)
DCG_EYE_Neuro-ophthmalogic_05	248,259	$647	($599, $695)
DCG_EYE_Neuro-ophthmalogic_07	33,369	$392	($263, $521)
DCG_EYE_vitreo-retinal_01	11,465	$27,761	($27,525, $27,997)
DCG_EYE_vitreo-retinal_02	24,803	$6,904	($6,753, $7,055)
DCG_EYE_vitreo-retinal_03	61,755	$3,917	($3,821, $4,014)
DCG_EYE_vitreo-retinal_04	22,268	$2,118	($1,960, $2,277)
DCG_EYE_vitreo-retinal_06	1,128,423	$559	($534, $583)
DCG_EYE_vitreo-retinal_07	2,023	$559	($534, $583)
DCG_EYE_Vision_impairment_01	6,441	$4,618	($4,323, $4,913)
DCG_EYE_Vision_impairment_02	39,892	$1,473	($1,354, $1,591)
DCG_EYE_Vision_impairment_03	133,877	$679	($614, $744)
DCG_EYE_Intraop_postproc_01	10,992	$3,611	($3,385, $3,837)
DCG_GEN_Breast_01	19,673	$25,060	($24,887, $25,233)

© 2024 Andriola C et al. *JAMA Health Forum*.

	Development Sample	DCG WLS Total Healthcare Spending Top-Coded at 250 K<br>K = 624<br>R-square in Validation Sample = 53.45%	
**Independent Variable Label**	**N**	**Coefficient**	**95% Confidence Interval**
DCG_GEN_Breast_02	10,925	$9,794	($9,568, $10,020)
DCG_GEN_Breast_03	112,743	$4,436	($4,366, $4,507)
DCG_GEN_Breast_04	103,582	$1,461	($1,387, $1,534)
DCG_GEN_Breast_05	164,870	$822	($764, $881)
DCG_GEN_Female_genital_01	135,535	$7,591	($7,526, $7,657)
DCG_GEN_Female_genital_02	134,001	$7,431	($7,372, $7,490)
DCG_GEN_Female_genital_03	33,007	$7,431	($7,372, $7,490)
DCG_GEN_Female_genital_04	3,539	$5,850	($5,453, $6,247)
DCG_GEN_Female_genital_05	170,531	$3,403	($3,344, $3,461)
DCG_GEN_Female_genital_06	251,482	$2,047	($1,999, $2,095)
DCG_GEN_Female_genital_07	533,532	$975	($942, $1,008)
DCG_GEN_Female_genital_08	9,783	$886	($645, $1,126)
DCG_GEN_Female_genital_09	313,767	$338	($294, $382)
DCG_GEN_Female_genital_10	758,301	$220	($192, $248)
DCG_GEN_Female_genital_prolapse_01	122,106	$2,398	($2,329, $2,466)
DCG_GEN_Male_genital_01	186,811	$7,380	($7,325, $7,435)
DCG_GEN_Male_genital_02	26,774	$3,051	($2,907, $3,196)
DCG_GEN_Male_genital_03	423,699	$1,025	($988, $1,063)
DCG_GEN_Male_genital_04	435,231	$487	($450, $523)
DCG_GEN_Male_genital_05	19,621	$459	($426, $493)
DCG_GEN_Male_genital_06	483,636	$459	($426, $493)
DCG_GEN_Urinary_01	41,377	$52,428	($52,288, $52,568)
DCG_GEN_Urinary_02	130,775	$10,894	($10,823, $10,964)
DCG_GEN_Urinary_03	44,071	$5,346	($5,229, $5,464)
DCG_GEN_Urinary_04	344,501	$2,903	($2,862, $2,944)
DCG_GEN_Urinary_05	275,296	$1,098	($1,068, $1,128)
DCG_GEN_Urinary_06	3,735	$1,098	($1,068, $1,128)
DCG_GEN_Urinary_07	54,485	$1,098	($1,068, $1,128)
DCG_GEN_Urinary_08	325,272	$1,098	($1,068, $1,128)
DCG_GEN_Urinary_09	1,166,562	$418	($396, $441)
DCG_GEN_Urinary_10	284,992	$191	($147, $236)
DCG_GEN_Intraop_postproc_01	3,194	$11,971	($11,553, $12,389)
DCG_GEN_Intraop_postproc_02	23,959	$7,268	($7,114, $7,422)
DCG_GEN_Symptoms_01	42,750	$8,150	($8,035, $8,264)
DCG_GEN_Symptoms_02	3,429	$2,963	($2,560, $3,366)
DCG_GEN_Symptoms_03	98,534	$2,175	($2,099, $2,252)
DCG_GEN_Symptoms_04	2,161	$1,068	($561, $1,576)
DCG_GEN_Symptoms_05	80,622	$797	($713, $881)
DCG_INF_Bacterial_other_01	50,938	$10,316	($10,208, $10,424)
DCG_INF_Bacterial_other_02	3,036	$4,903	($4,474, $5,331)

© 2024 Andriola C et al. *JAMA Health Forum*.

	Development Sample	DCG WLS Total Healthcare Spending Top-Coded at 250 K<br>K = 624<br>R-square in Validation Sample = 53.45%	
**Independent Variable Label**	**N**	**Coefficient**	**95% Confidence Interval**
DCG_INF_Bacterial_other_03	41,540	$2,634	($2,518, $2,750)
DCG_INF_Bacterial_other_04	5,300	$939	($615, $1,263)
DCG_INF_Bacterial_other_05	307,126	$352	($309, $395)
DCG_INF_Staph_strep_01	70,998	$8,307	($8,215, $8,398)
DCG_INF_Staph_strep_02	40,972	$2,736	($2,619, $2,854)
DCG_INF_Mycobacterial_01	11,403	$4,485	($4,262, $4,708)
DCG_INF_Mycobacterial_02	37,062	$1,444	($1,321, $1,567)
DCG_INF_Mycoses_01	4,504	$16,293	($15,939, $16,648)
DCG_INF_Mycoses_02	11,373	$5,663	($5,442, $5,885)
DCG_INF_Mycoses_03	4,024	$4,375	($4,003, $4,748)
DCG_INF_Mycoses_04	8,853	$1,050	($799, $1,301)
DCG_INF_Mycoses_05	677,949	$637	($607, $666)
DCG_INF_Parasitic_01	363,354	$236	($196, $276)
DCG_INF_Viral_other_01	6,251	$12,362	($12,061, $12,662)
DCG_INF_Viral_other_02	2,406	$1,518	($1,037, $1,999)
DCG_INF_Viral_other_03	1,827,349	$159	($141, $177)
DCG_INF_Coronavirus_01	2,298	$11,423	($10,930, $11,915)
DCG_INF_HIV_01	87,550	$28,945	($28,864, $29,026)
DCG_INF_Viral_hepatitis_01	59,559	$17,387	($17,289, $17,485)
DCG_INF_Viral_hepatitis_02	41,692	$4,043	($3,928, $4,159)
DCG_INF_Viral_hepatitis_03	8,497	$2,691	($2,434, $2,948)
DCG_INF_Gastrointestinal_infection_02	28,202	$9,600	($9,458, $9,742)
DCG_INF_Gastrointestinal_infection_03	5,743	$2,998	($2,686, $3,309)
DCG_INF_Gastrointestinal_infection_04	3,320	$1,389	($979, $1,799)
DCG_INF_Sexually_transmitted_01	22,920	$2,432	($2,275, $2,588)
DCG_INF_Sexually_transmitted_02	9,960	$1,794	($1,554, $2,033)
DCG_INF_Sexually_transmitted_03	250,469	$326	($278, $374)
DCG_INF_Sepsis_01	5,883	$22,249	($21,887, $22,611)
DCG_INF_Sepsis_02	124,784	$13,340	($13,263, $13,416)
DCG_INF_CNS_01	2,316	$6,965	($6,467, $7,462)
DCG_INF_Symptoms_01	9,142	$10,726	($10,479, $10,974)
DCG_INF_Symptoms_02	30,109	$898	($762, $1,034)
DCG_INJ_Head_neck_eye_01	8,063	$22,606	($22,403, $22,809)
DCG_INJ_Head_neck_eye_02	5,852	$22,606	($22,403, $22,809)
DCG_INJ_Head_neck_eye_03	4,569	$16,447	($16,094, $16,800)
DCG_INJ_Head_neck_eye_04	13,829	$9,762	($9,561, $9,963)
DCG_INJ_Head_neck_eye_05	48,286	$3,849	($3,741, $3,956)
DCG_INJ_Head_neck_eye_06	35,470	$2,795	($2,669, $2,921)
DCG_INJ_Head_neck_eye_07	412,723	$1,007	($970, $1,045)
DCG_INJ_Head_neck_eye_08	11,994	$795	($638, $951)

© 2024 Andriola C et al. *JAMA Health Forum*.

	Development Sample	DCG WLS Total Healthcare Spending Top-Coded at 250 K<br>K = 624<br>R-square in Validation Sample = 53.45%	
**Independent Variable Label**	**N**	**Coefficient**	**95% Confidence Interval**
DCG_INJ_Head_neck_eye_09	10,812	$795	($638, $951)
DCG_INJ_Head_neck_eye_10	26,908	$481	($336, $626)
DCG_INJ_Abdominal_01	12,474	$16,876	($16,662, $17,091)
DCG_INJ_Abdominal_02	2,101	$5,337	($4,822, $5,852)
DCG_INJ_Spine_back_01	9,343	$19,185	($18,936, $19,435)
DCG_INJ_Spine_back_02	33,950	$9,933	($9,800, $10,065)
DCG_INJ_Spine_back_03	7,014	$5,180	($4,898, $5,463)
DCG_INJ_Spine_back_04	236,711	$2,443	($2,394, $2,492)
DCG_INJ_Fracture_01	27,510	$20,339	($20,195, $20,482)
DCG_INJ_Fracture_02	60,883	$5,781	($5,684, $5,878)
DCG_INJ_Fracture_03	32,952	$5,723	($5,593, $5,853)
DCG_INJ_Fracture_04	455,195	$2,489	($2,453, $2,524)
DCG_INJ_Fracture_05	334,542	$1,611	($1,570, $1,652)
DCG_INJ_Fracture_06	423,366	$361	($324, $398)
DCG_INJ_Minor_01	35,041	$16,734	($16,606, $16,862)
DCG_INJ_Minor_02	42,455	$8,462	($8,351, $8,573)
DCG_INJ_Minor_03	3,542	$8,462	($8,351, $8,573)
DCG_INJ_Minor_04	15,654	$4,482	($4,290, $4,674)
DCG_INJ_Minor_05	195,913	$2,617	($2,563, $2,671)
DCG_INJ_Minor_06	1,404,861	$1,249	($1,229, $1,270)
DCG_INJ_Minor_07	209,212	$644	($593, $696)
DCG_INJ_Minor_08	2,577,418	$284	($268, $299)
DCG_INJ_Minor_09	17,902	$284	($268, $299)
DCG_INJ_Foreign_body_01	208,205	$1,372	($1,320, $1,424)
DCG_INJ_Foreign_body_02	66,438	$598	($506, $690)
DCG_INJ_Burn_01	2,795	$8,002	($7,551, $8,453)
DCG_INJ_Burn_02	11,743	$1,915	($1,698, $2,133)
DCG_INJ_Burn_03	62,831	$584	($490, $678)
DCG_INJ_Poisoning_01	37,891	$17,003	($16,872, $17,134)
DCG_INJ_Poisoning_02	19,629	$7,255	($7,086, $7,425)
DCG_INJ_Poisoning_03	135,078	$2,796	($2,730, $2,862)
DCG_INJ_Poisoning_04	53,757	$1,541	($1,438, $1,644)
DCG_INJ_Poisoning_05	3,960	$810	($435, $1,185)
DCG_INJ_Abuse_01	5,762	$1,969	($1,659, $2,280)
DCG_INJ_Abuse_02	20,245	$648	($482, $814)
DCG_INJ_Allergies_01	105,599	$1,351	($1,278, $1,424)
DCG_INJ_Allergies_02	6,042,515	$43	($32, $54)
DCG_INJ_Complic_01	38,789	$37,712	($37,582, $37,841)
DCG_INJ_Complic_02	89,187	$18,323	($18,242, $18,404)
DCG_INJ_Complic_03	43,023	$5,423	($5,309, $5,537)

© 2024 Andriola C et al. *JAMA Health Forum*.

	Development Sample	DCG WLS Total Healthcare Spending Top-Coded at 250 K<br>K = 624<br>R-square in Validation Sample = 53.45%	
**Independent Variable Label**	**N**	**Coefficient**	**95% Confidence Interval**
DCG_INJ_Complic_04	2,607	$1,993	($1,531, $2,455)
DCG_INJ_Nerves_01	374,482	$3,374	($3,333, $3,415)
DCG_INJ_Nerves_02	61,110	$1,082	($985, $1,178)
DCG_INJ_Traumatic_injuries_01	9,210	$6,472	($6,225, $6,719)
DCG_INJ_Traumatic_injuries_02	4,400	$1,152	($796, $1,508)
DCG_INJ_Traumatic_injuries_03	28,083	$420	($279, $561)
DCG_INJ_Vascular_01	8,073	$11,725	($11,462, $11,989)
DCG_MAL_Face_01	35,141	$4,095	($3,963, $4,226)
DCG_MAL_Face_02	67,509	$1,598	($1,505, $1,691)
DCG_MAL_Circulatory_01	70,120	$10,654	($10,563, $10,745)
DCG_MAL_Circulatory_02	146,664	$3,897	($3,834, $3,960)
DCG_MAL_Musculoskeletal_01	5,276	$7,997	($7,668, $8,326)
DCG_MAL_Musculoskeletal_02	308,589	$1,908	($1,864, $1,951)
DCG_MAL_Neurologic_01	38,175	$5,674	($5,548, $5,799)
DCG_MAL_Neurologic_02	15,198	$3,155	($2,961, $3,348)
DCG_MAL_Internal_01	6,459	$11,077	($10,781, $11,373)
DCG_MAL_Internal_02	40,273	$5,061	($4,943, $5,179)
DCG_MAL_Internal_03	97,643	$2,288	($2,207, $2,370)
DCG_MAL_Internal_04	42,849	$717	($612, $823)
DCG_MAL_Internal_05	11,896	$717	($612, $823)
DCG_MAL_Other_01	230,449	$1,832	($1,782, $1,883)
DCG_MAL_Other_02	13,155	$856	($635, $1,078)
DCG_MBD_Anxiety_01	5,738	$9,245	($8,934, $9,556)
DCG_MBD_Anxiety_02	31,402	$5,088	($4,953, $5,223)
DCG_MBD_Anxiety_03	136,242	$1,961	($1,897, $2,026)
DCG_MBD_Anxiety_04	3,575,171	$600	($586, $615)
DCG_MBD_Dementia_01	6,775	$1,375	($1,086, $1,665)
DCG_MBD_Eating_Disorder_01	79,840	$5,231	($5,147, $5,315)
DCG_MBD_Eating_Disorder_02	10,832	$2,022	($1,817, $2,228)
DCG_MBD_Eating_Disorder_03	2,360	$2,022	($1,817, $2,228)
DCG_MBD_Gender_Sexuality_01	21,741	$4,239	($4,078, $4,399)
DCG_MBD_Mood_Disorder_01	23,828	$9,438	($9,282, $9,594)
DCG_MBD_Mood_Disorder_02	388,548	$3,770	($3,730, $3,809)
DCG_MBD_Mood_Disorder_03	2,661,217	$1,223	($1,206, $1,239)
DCG_MBD_Mood_Disorder_04	13,453	$478	($273, $682)
DCG_MBD_Mood_Disorder_05	72,354	$262	($174, $349)
DCG_MBD_Neuro_Physio_Develop_01	11,243	$9,686	($9,462, $9,910)
DCG_MBD_Neuro_Physio_Develop_02	7,005	$7,427	($7,144, $7,709)
DCG_MBD_Neuro_Physio_Develop_03	253,888	$4,793	($4,745, $4,841)
DCG_MBD_Neuro_Physio_Develop_04	34,327	$3,358	($3,229, $3,487)

© 2024 Andriola C et al. *JAMA Health Forum*.

	Development Sample	DCG WLS Total Healthcare Spending Top-Coded at 250 K<br>K = 624<br>R-square in Validation Sample = 53.45%	
**Independent Variable Label**	**N**	**Coefficient**	**95% Confidence Interval**
DCG_MBD_Neuro_Physio_Develop_05	12,547	$1,528	($1,317, $1,740)
DCG_MBD_Neuro_Physio_Develop_06	1,519,058	$1,137	($1,117, $1,157)
DCG_MBD_Neuro_Physio_Develop_07	170,864	$459	($401, $517)
DCG_MBD_Personality_Behavioral_Other_01	16,481	$9,418	($9,234, $9,602)
DCG_MBD_Personality_Behavioral_Other_02	40,659	$4,848	($4,729, $4,967)
DCG_MBD_Personality_Behavioral_Other_03	22,745	$1,671	($1,544, $1,798)
DCG_MBD_Personality_Behavioral_Other_04	12,006	$1,671	($1,544, $1,798)
DCG_MBD_Psychosis_01	22,850	$9,004	($8,843, $9,166)
DCG_MBD_Psychosis_02	11,916	$3,845	($3,627, $4,063)
DCG_MBD_Psychosis_03	101,019	$266	($191, $340)
DCG_MBD_Schizophrenia_01	16,198	$3,572	($3,383, $3,760)
DCG_MBD_Sleep_01	6,259	$620	($322, $919)
DCG_MBD_Stress_Trauma_01	6,428	$5,754	($5,459, $6,049)
DCG_MBD_Stress_Trauma_02	223,628	$2,387	($2,336, $2,438)
DCG_MBD_Stress_Trauma_03	1,011,933	$1,526	($1,502, $1,550)
DCG_MBD_Stress_Trauma_04	55,338	$583	($482, $683)
DCG_MBD_Stress_Trauma_05	188,042	$370	($314, $425)
DCG_MBD_Substance_Abuse_01	49,078	$24,382	($24,275, $24,490)
DCG_MBD_Substance_Abuse_02	254,898	$6,007	($5,959, $6,055)
DCG_MBD_Substance_Abuse_03	20,726	$2,959	($2,874, $3,044)
DCG_MBD_Substance_Abuse_04	57,477	$2,959	($2,874, $3,044)
DCG_MBD_Substance_Abuse_05	42,784	$1,975	($1,861, $2,090)
DCG_MBD_Substance_Abuse_06	1,058,895	$1,253	($1,229, $1,277)
DCG_MBD_Substance_Abuse_07	19,400	$1,008	($839, $1,178)
DCG_MBD_Suicide_01	117,946	$7,994	($7,921, $8,067)
DCG_MBD_Symptoms_01	7,243	$1,764	($1,486, $2,041)
DCG_MSK_Arthritides_01	242,867	$15,473	($15,423, $15,522)
DCG_MSK_Arthritides_02	64,885	$10,368	($10,131, $10,606)
DCG_MSK_Arthritides_03	16,093	$8,411	($8,225, $8,597)
DCG_MSK_Arthritides_04	4,724	$6,399	($6,023, $6,775)
DCG_MSK_Arthritides_05	18,172	$4,089	($3,913, $4,264)
DCG_MSK_Arthritides_06	40,572	$2,760	($2,642, $2,878)
DCG_MSK_Arthritides_07	494,724	$515	($480, $549)
DCG_MSK_Arthritides_08	159,261	$439	($378, $501)
DCG_MSK_Bone_metab_01	25,999	$13,336	($13,187, $13,485)
DCG_MSK_Bone_metab_02	1,352,082	$3,457	($3,435, $3,479)
DCG_MSK_Bone_metab_03	2,961	$3,457	($3,435, $3,479)
DCG_MSK_Bone_metab_04	620,745	$1,078	($1,048, $1,109)
DCG_MSK_Bone_metab_05	281,896	$551	($506, $596)
DCG_MSK_Bone_metab_06	165,817	$481	($422, $539)

© 2024 Andriola C et al. *JAMA Health Forum*.

	Development Sample	DCG WLS Total Healthcare Spending Top-Coded at 250 K<br>K = 624<br>R-square in Validation Sample = 53.45%	
**Independent Variable Label**	**N**	**Coefficient**	**95% Confidence Interval**
DCG_MSK_Bone_metab_07	134,703	$220	($155, $284)
DCG_MSK_Fractures_01	11,295	$5,598	($5,370, $5,827)
DCG_MSK_Fractures_02	16,273	$5,151	($4,964, $5,338)
DCG_MSK_Fractures_03	64,905	$853	($760, $946)
DCG_MSK_Infect_01	32,163	$14,373	($14,235, $14,512)
DCG_MSK_Infect_02	14,167	$6,916	($6,677, $7,154)
DCG_MSK_Infect_03	6,184	$3,347	($3,047, $3,647)
DCG_MSK_Infect_04	11,689	$1,370	($1,151, $1,588)
DCG_MSK_Soft_tissue_disorders_01	5,688	$17,682	($17,369, $17,995)
DCG_MSK_Soft_tissue_disorders_02	52,521	$8,040	($7,936, $8,144)
DCG_MSK_Soft_tissue_disorders_03	931,640	$4,675	($4,648, $4,701)
DCG_MSK_Soft_tissue_disorders_04	549,461	$974	($942, $1,007)
DCG_MSK_Soft_tissue_disorders_06	3,206,910	$728	($713, $743)
DCG_MSK_Soft_tissue_disorders_07	305,000	$237	($210, $264)
DCG_MSK_Soft_tissue_disorders_08	513,992	$237	($210, $264)
DCG_MSK_Soft_tissue_disorders_09	5,853,303	$55	($44, $67)
DCG_MSK_Spine_disorders_01	24,264	$25,247	($25,094, $25,399)
DCG_MSK_Spine_disorders_02	395,743	$10,439	($10,400, $10,478)
DCG_MSK_Spine_disorders_03	296,216	$3,515	($3,471, $3,559)
DCG_MSK_Spine_disorders_04	2,095,079	$1,216	($1,198, $1,233)
DCG_MSK_Spine_disorders_05	33,113	$1,216	($1,198, $1,233)
DCG_MSK_Spine_disorders_06	1,274,129	$446	($424, $468)
DCG_MSK_Spine_disorders_07	74,958	$227	($141, $314)
DCG_MSK_Intraop_postproc_01	100,382	$10,491	($10,414, $10,568)
DCG_MSK_Symptoms_01	7,443	$3,008	($2,734, $3,282)
DCG_NEO_Ear_resp_thoracic_01	21,519	$30,027	($29,847, $30,206)
DCG_NEO_Ear_resp_thoracic_02	22,016	$22,313	($22,149, $22,478)
DCG_NEO_Ear_resp_thoracic_03	14,849	$7,339	($7,143, $7,535)
DCG_NEO_Ear_resp_thoracic_04	4,589	$4,151	($3,802, $4,499)
DCG_NEO_Breast_01	241,780	$11,926	($11,875, $11,977)
DCG_NEO_Breast_02	22,619	$3,332	($3,175, $3,490)
DCG_NEO_Breast_03	59,939	$2,301	($2,203, $2,399)
DCG_NEO_Breast_04	7,900	$740	($474, $1,005)
DCG_NEO_Digestive_system_01	4,111	$19,789	($19,700, $19,878)
DCG_NEO_Digestive_system_02	78,272	$19,789	($19,700, $19,878)
DCG_NEO_Digestive_system_03	3,398	$19,789	($19,700, $19,878)
DCG_NEO_Digestive_system_04	3,070	$14,058	($13,631, $14,485)
DCG_NEO_Digestive_system_05	105,116	$7,710	($7,509, $7,912)
DCG_NEO_Digestive_system_06	18,040	$4,896	($4,718, $5,074)
DCG_NEO_Digestive_system_07	4,952	$1,500	($1,158, $1,842)

© 2024 Andriola C et al. *JAMA Health Forum*.

	Development Sample	DCG WLS Total Healthcare Spending Top-Coded at 250 K<br>K = 624<br>R-square in Validation Sample = 53.45%	
**Independent Variable Label**	**N**	**Coefficient**	**95% Confidence Interval**
DCG_NEO_Digestive_system_08	749,683	$1,285	($1,253, $1,317)
DCG_NEO_Urinary_tract_01	20,521	$8,211	($8,114, $8,308)
DCG_NEO_Urinary_tract_02	36,565	$8,211	($8,114, $8,308)
DCG_NEO_Urinary_tract_03	3,143	$8,211	($8,114, $8,308)
DCG_NEO_Urinary_tract_04	21,195	$3,371	($3,208, $3,533)
DCG_NEO_Blood_lymph_tissues_01	12,449	$66,790	($66,575, $67,005)
DCG_NEO_Blood_lymph_tissues_02	14,917	$59,111	($58,915, $59,306)
DCG_NEO_Blood_lymph_tissues_03	68,546	$20,073	($19,998, $20,148)
DCG_NEO_Blood_lymph_tissues_04	51,926	$20,073	($19,998, $20,148)
DCG_NEO_Blood_lymph_tissues_05	12,245	$7,129	($6,915, $7,342)
DCG_NEO_Blood_lymph_tissues_06	12,096	$2,310	($2,094, $2,527)
DCG_NEO_Lip_mouth_throat_01	13,768	$9,931	($9,728, $10,134)
DCG_NEO_Lip_mouth_throat_02	7,754	$6,451	($6,181, $6,720)
DCG_NEO_Lip_mouth_throat_03	29,274	$2,460	($2,329, $2,592)
DCG_NEO_Lip_mouth_throat_04	3,968	$2,460	($2,329, $2,592)
DCG_NEO_Lip_mouth_throat_05	3,630	$1,963	($1,567, $2,358)
DCG_NEO_Genital_female_01	89,615	$6,686	($6,606, $6,766)
DCG_NEO_Genital_female_02	23,243	$5,879	($5,724, $6,034)
DCG_NEO_Genital_female_03	358,581	$2,468	($2,425, $2,511)
DCG_NEO_Genital_female_04	6,469	$2,032	($1,738, $2,325)
DCG_NEO_Genital_male_01	111,698	$880	($685, $1,075)
DCG_NEO_Genital_male_02	8,238	$715	($455, $975)
DCG_NEO_Brain_CNS_01	27,230	$27,320	($27,185, $27,456)
DCG_NEO_Brain_CNS_02	8,143	$27,320	($27,185, $27,456)
DCG_NEO_Brain_CNS_03	13,956	$5,530	($5,329, $5,730)
DCG_NEO_Periph_nerves_autonomic_01	3,228	$8,686	($8,265, $9,106)
DCG_NEO_Endocrine_01	16,757	$24,539	($24,342, $24,736)
DCG_NEO_Endocrine_02	2,820	$16,638	($16,186, $17,089)
DCG_NEO_Endocrine_03	157,014	$2,557	($2,493, $2,620)
DCG_NEO_Skin_01	2,196	$30,022	($29,513, $30,530)
DCG_NEO_Skin_02	40,256	$6,580	($6,462, $6,699)
DCG_NEO_Skin_03	45,833	$2,898	($2,788, $3,009)
DCG_NEO_Skin_04	223,396	$1,346	($1,295, $1,397)
DCG_NEO_Skin_05	31,992	$1,148	($1,016, $1,281)
DCG_NEO_Skin_06	124,537	$607	($538, $675)
DCG_NEO_Skin_07	1,109,726	$268	($244, $292)
DCG_NEO_Mesenchymal_01	25,306	$39,042	($38,871, $39,213)
DCG_NEO_Mesenchymal_02	19,130	$16,886	($16,713, $17,059)
DCG_NEO_Mesenchymal_03	50,673	$3,678	($3,572, $3,784)
DCG_NEO_Mesenchymal_04	24,368	$2,719	($2,567, $2,870)

© 2024 Andriola C et al. *JAMA Health Forum*.

	Development Sample	DCG WLS Total Healthcare Spending Top-Coded at 250 K<br>K = 624<br>R-square in Validation Sample = 53.45%	
**Independent Variable Label**	**N**	**Coefficient**	**95% Confidence Interval**
DCG_NEO_Mesenchymal_06	132,556	$403	($338, $468)
DCG_NEO_Peritoneum_01	2,811	$14,221	($13,768, $14,675)
DCG_NEO_Eye_01	4,985	$5,525	($5,189, $5,861)
DCG_NEO_Other_01	150,220	$18,943	($18,864, $19,022)
DCG_NEO_Other_02	10,910	$10,199	($9,972, $10,426)
DCG_NEO_Other_03	4,592,582	$320	($305, $335)
DCG_NEO_Symptoms_01	3,025	$964	($535, $1,393)
DCG_NVS_Epilepsy_01	3,313	$43,026	($42,612, $43,439)
DCG_NVS_Epilepsy_02	29,705	$10,496	($10,358, $10,634)
DCG_NVS_Epilepsy_03	210,013	$4,763	($4,710, $4,815)
DCG_NVS_Epilepsy_04	89,833	$3,233	($3,154, $3,312)
DCG_NVS_Headache_01	24,801	$4,548	($4,398, $4,698)
DCG_NVS_Headache_02	184,252	$3,604	($3,548, $3,659)
DCG_NVS_Headache_03	8,017	$2,614	($2,351, $2,878)
DCG_NVS_Headache_04	717,221	$1,092	($1,063, $1,120)
DCG_NVS_Headache_05	2,040,709	$307	($290, $325)
DCG_NVS_Sleep disorders_01	30,661	$13,600	($13,465, $13,735)
DCG_NVS_Sleep disorders_02	1,414,555	$2,719	($2,697, $2,740)
DCG_NVS_Sleep disorders_03	6,735	$1,265	($977, $1,553)
DCG_NVS_Sleep disorders_04	900,381	$556	($531, $582)
DCG_NVS_Sleep disorders_05	191,267	$262	($208, $316)
DCG_NVS_Movement disorders_01	22,309	$7,013	($6,866, $7,160)
DCG_NVS_Movement disorders_02	3,703	$7,013	($6,866, $7,160)
DCG_NVS_Movement disorders_03	15,135	$4,836	($4,640, $5,032)
DCG_NVS_Movement disorders_04	2,096	$3,376	($2,860, $3,893)
DCG_NVS_Movement disorders_05	16,373	$2,325	($2,136, $2,513)
DCG_NVS_Neuropathies_01	10,020	$28,715	($28,479, $28,951)
DCG_NVS_Neuropathies_02	5,852	$13,836	($13,527, $14,146)
DCG_NVS_Neuropathies_03	17,280	$10,994	($10,807, $11,181)
DCG_NVS_Neuropathies_04	23,633	$4,670	($4,516, $4,824)
DCG_NVS_Neuropathies_05	724,988	$1,936	($1,906, $1,965)
DCG_NVS_Neuropathies_06	10,299	$1,486	($1,254, $1,719)
DCG_NVS_Neuropathies_07	51,236	$747	($643, $852)
DCG_NVS_Neuropathies_08	358,670	$435	($395, $475)
DCG_NVS_Myoneural_junc_musc_01	13,073	$12,704	($12,496, $12,911)
DCG_NVS_Myoneural_junc_musc_02	18,580	$5,833	($5,659, $6,007)
DCG_NVS_Myoneural_junc_musc_03	2,742	$3,537	($3,087, $3,988)
DCG_NVS_Pain_syndromes_01	25,367	$26,551	($26,391, $26,711)
DCG_NVS_Pain_syndromes_02	1,283,865	$7,467	($7,444, $7,489)
DCG_NVS_Pain_syndromes_03	179,547	$2,374	($2,317, $2,431)

© 2024 Andriola C et al. *JAMA Health Forum*.

	Development Sample	DCG WLS Total Healthcare Spending Top-Coded at 250 K<br>K = 624<br>R-square in Validation Sample = 53.45%	
**Independent Variable Label**	**N**	**Coefficient**	**95% Confidence Interval**
DCG_NVS_Infection_and_inflammatory_CNS_01	92,655	$49,129	($49,050, $49,208)
DCG_NVS_Infection_and_inflammatory_CNS_02	3,109	$28,125	($27,697, $28,552)
DCG_NVS_Infection_and_inflammatory_CNS_03	6,165	$19,066	($18,762, $19,370)
DCG_NVS_Infection_and_inflammatory_CNS_04	8,501	$13,273	($13,016, $13,530)
DCG_NVS_Infection_and_inflammatory_CNS_05	5,854	$4,726	($4,417, $5,035)
DCG_NVS_Infection_and_inflammatory_CNS_06	11,777	$2,249	($2,027, $2,471)
DCG_NVS_Stroke_01	79,131	$2,805	($2,718, $2,891)
DCG_NVS_Stroke_02	3,191	$1,250	($829, $1,672)
DCG_NVS_Coma_01	9,313	$63,396	($63,137, $63,656)
DCG_NVS_Coma_02	15,535	$15,228	($15,030, $15,425)
DCG_NVS_Coma_03	14,560	$6,776	($6,574, $6,977)
DCG_NVS_Coma_04	7,411	$4,413	($4,138, $4,687)
DCG_NVS_Coma_05	67,541	$2,364	($2,272, $2,455)
DCG_NVS_Degenerative_01	4,477	$5,361	($5,007, $5,716)
DCG_NVS_Degenerative_02	342,569	$1,098	($1,058, $1,138)
DCG_NVS_Degenerative_03	71,437	$1,098	($1,058, $1,138)
DCG_NVS_Atrophies_motor_neural_disease_01	4,701	$21,852	($21,504, $22,199)
DCG_NVS_Atrophies_motor_neural_disease_02	400,915	$4,051	($4,009, $4,094)
DCG_NVS_Atrophies_motor_neural_disease_03	3,715	$2,328	($1,938, $2,718)
DCG_NVS_CP_oth_paralytic_01	7,507	$20,523	($20,242, $20,805)
DCG_NVS_CP_oth_paralytic_02	29,497	$16,843	($16,698, $16,989)
DCG_NVS_CP_oth_paralytic_03	19,051	$8,885	($8,710, $9,061)
DCG_NVS_CP_oth_paralytic_04	29,083	$5,522	($5,381, $5,663)
DCG_NVS_CP_oth_paralytic_05	10,211	$2,145	($1,892, $2,398)
DCG_NVS_intraop_postproc_01	14,558	$17,804	($17,607, $18,001)
DCG_NVS_Symptoms_01	23,815	$4,029	($3,876, $4,183)
DCG_NVS_Symptoms_02	259,291	$2,257	($2,210, $2,304)
DCG_NVS_Symptoms_03	39,101	$1,645	($1,526, $1,765)
DCG_NVS_Symptoms_04	18,819	$1,188	($1,015, $1,360)
DCG_NVS_Symptoms_05	100,783	$170	($96, $245)
DCG_NVS_Symptoms_06	509,024	$72	($38, $105)
DCG_PNL_Infection_01	6,068	$5,786	($5,479, $6,093)
DCG_PNL_BirthTrauma_01	3,802	$12,415	($12,022, $12,808)
DCG_PNL_BirthTrauma_03	23,038	$1,040	($867, $1,213)
DCG_PNL_GestationLength_01	55,585	$14,950	($14,834, $15,065)
DCG_PNL_GestationLength_02	14,445	$3,270	($3,071, $3,468)

© 2024 Andriola C et al. *JAMA Health Forum*.

	Development Sample	DCG WLS Total Healthcare Spending Top-Coded at 250 K<br>K = 624<br>R-square in Validation Sample = 53.45%	
**Independent Variable Label**	**N**	**Coefficient**	**95% Confidence Interval**
DCG_PNL_MaternalConditions_01	15,604	$7,054	($6,857, $7,250)
DCG_PNL_MaternalConditions_02	103,908	$1,858	($1,770, $1,946)
DCG_PNL_Hematological_01	8,488	$39,655	($39,374, $39,936)
DCG_PNL_Hematological_02	5,559	$1,705	($1,530, $1,880)
DCG_PNL_Hematological_03	13,260	$1,705	($1,530, $1,880)
DCG_PNL_Hematological_04	152,141	$486	($417, $555)
DCG_PNL_Respiratory_01	26,366	$34,632	($34,464, $34,800)
DCG_PNL_Respiratory_02	20,354	$9,695	($9,523, $9,868)
DCG_PNL_Neurological_01	5,067	$11,646	($11,309, $11,983)
DCG_PNL_Neurological_02	5,529	$5,475	($5,156, $5,795)
DCG_PNL_Other_01	13,274	$14,632	($14,418, $14,846)
DCG_PNL_Other_02	41,862	$5,154	($5,030, $5,278)
DCG_PNL_Other_03	6,794	$1,949	($1,659, $2,239)
DCG_PNL_Other_04	144,533	$1,023	($953, $1,093)
DCG_PNL_Other_05	19,643	$475	($304, $646)
DCG_PRG_Glucose_Intolerance_01	67,487	$3,862	($3,766, $3,957)
DCG_PRG_Glucose_Intolerance_02	5,976	$1,404	($1,098, $1,710)
DCG_PRG_Hypertension_01	19,352	$6,982	($6,808, $7,155)
DCG_PRG_Hypertension_02	19,691	$3,537	($3,366, $3,708)
DCG_PRG_Hypertension_03	24,227	$2,172	($2,017, $2,327)
DCG_PRG_Hypertension_04	32,802	$1,831	($1,696, $1,965)
DCG_PRG_Multiple_Gestation_01	18,920	$9,098	($8,923, $9,273)
DCG_PRG_Abortive_01	13,541	$4,794	($4,586, $5,001)
DCG_PRG_Abortive_02	61,304	$3,505	($3,405, $3,604)
DCG_PRG_Abortive_03	13,815	$1,612	($1,410, $1,813)
DCG_PRG_Abortive_04	8,201	$1,297	($1,034, $1,560)
DCG_PRG_Fetal_Complications_01	20,353	$6,348	($6,178, $6,518)
DCG_PRG_Fetal_Complications_02	13,047	$4,714	($4,504, $4,925)
DCG_PRG_Fetal_Complications_03	51,337	$3,703	($3,593, $3,813)
DCG_PRG_Fetal_Complications_04	49,691	$2,344	($2,234, $2,455)
DCG_PRG_Fetal_Complications_05	18,976	$756	($582, $929)
DCG_PRG_Maternal_Complications_01	13,712	$11,135	($10,929, $11,341)
DCG_PRG_Maternal_Complications_02	255,143	$2,688	($2,631, $2,746)
DCG_PRG_Maternal_Complications_03	3,975	$1,222	($847, $1,597)
DCG_PRG_Maternal_Complications_04	2,468	$1,155	($680, $1,631)
DCG_PRG_Maternal_Complications_05	14,058	$654	($453, $855)
DCG_PRG_Delivery_01	396,552	$11,023	($10,974, $11,071)
DCG_PRG_Delivery_02	40,456	$9,646	($9,524, $9,767)
DCG_PRG_Delivery_03	13,179	$8,681	($8,469, $8,892)
DCG_PRG_Delivery_04	2,032	$8,103	($7,578, $8,627)

© 2024 Andriola C et al. *JAMA Health Forum*.

	Development Sample	DCG WLS Total Healthcare Spending Top-Coded at 250 K<br>K = 624<br>R-square in Validation Sample = 53.45%	
**Independent Variable Label**	**N**	**Coefficient**	**95% Confidence Interval**
DCG_PRG_Delivery_05	10,004	$5,208	($4,969, $5,448)
DCG_PRG_Delivery_06	273,800	$1,199	($1,149, $1,249)
DCG_PRG_Placenta_01	42,972	$4,311	($4,192, $4,430)
DCG_PRG_Placenta_02	17,521	$2,585	($2,405, $2,766)
DCG_PRG_Placenta_03	100,342	$1,076	($996, $1,156)
DCG_PRG_Postpartum_01	70,534	$8,250	($8,158, $8,343)
DCG_PRG_Other_01	110,332	$5,290	($5,213, $5,366)
DCG_PRG_Other_02	2,292	$2,522	($2,029, $3,016)
DCG_PRG_Other_03	57,830	$1,565	($1,463, $1,667)
DCG_PRG_Other_04	20,529	$1,078	($911, $1,245)
DCG_RSP_Fail_01	108,474	$29,080	($28,999, $29,161)
DCG_RSP_Fail_02	8,273	$12,750	($12,488, $13,011)
DCG_RSP_Pneumonia_Influenza_01	16,462	$12,380	($12,190, $12,570)
DCG_RSP_Pneumonia_Influenza_02	12,865	$11,630	($11,418, $11,842)
DCG_RSP_Pneumonia_Influenza_03	15,783	$4,214	($4,026, $4,403)
DCG_RSP_Pneumonia_Influenza_04	565,770	$1,026	($994, $1,058)
DCG_RSP_Lower_01	6,686	$12,480	($12,187, $12,773)
DCG_RSP_Lower_02	18,602	$7,025	($6,849, $7,201)
DCG_RSP_Lower_03	2,610,487	$1,543	($1,528, $1,559)
DCG_RSP_Interstitium_01	6,286	$8,067	($7,769, $8,365)
DCG_RSP_Upper_01	15,076	$10,293	($10,100, $10,486)
DCG_RSP_Upper_02	67,181	$6,211	($6,120, $6,303)
DCG_RSP_Upper_03	635,023	$2,146	($2,115, $2,176)
DCG_RSP_Upper_04	1,007,526	$432	($408, $456)
DCG_RSP_Other_01	28,487	$26,718	($26,576, $26,861)
DCG_RSP_Other_02	69,815	$17,077	($16,983, $17,171)
DCG_RSP_Other_03	3,594	$5,008	($4,614, $5,401)
DCG_RSP_Other_04	9,160	$797	($551, $1,044)
DCG_RSP_Symptoms_01	10,557	$11,738	($11,508, $11,968)
DCG_RSP_Symptoms_02	109,670	$3,214	($3,142, $3,285)
DCG_RSP_Symptoms_03	429,774	$2,112	($2,075, $2,149)
DCG_RSP_Symptoms_04	887,407	$971	($946, $997)
DCG_RSP_Symptoms_05	58,466	$621	($524, $719)
DCG_RSP_Symptoms_06	487,654	$93	($59, $126)
DCG_SKN_Benign_neo_skin_01	48,105	$1,774	($1,666, $1,882)
DCG_SKN_Benign_neo_skin_02	21,306	$744	($582, $905)
DCG_SKN_Benign_neo_skin_03	664,673	$243	($214, $273)
DCG_SKN_Inflamatory_dermatoses_01	75,634	$17,653	($17,432, $17,873)
DCG_SKN_Inflamatory_dermatoses_02	296,588	$7,782	($7,739, $7,826)
DCG_SKN_Inflamatory_dermatoses_03	9,201	$5,428	($5,120, $5,736)

© 2024 Andriola C et al. *JAMA Health Forum*.

	Development Sample	DCG WLS Total Healthcare Spending Top-Coded at 250 K<br>K = 624<br>R-square in Validation Sample = 53.45%	
**Independent Variable Label**	**N**	**Coefficient**	**95% Confidence Interval**
DCG_SKN_Inflamatory_dermatoses_04	36,520	$3,317	($3,086, $3,547)
DCG_SKN_Inflamatory_dermatoses_05	44,204	$1,874	($1,761, $1,987)
DCG_SKN_Inflamatory_dermatoses_06	3,728,834	$522	($508, $535)
DCG_SKN_Inflamatory_dermatoses_07	1,572,020	$218	($198, $237)
DCG_SKN_Infections_01	21,380	$4,522	($4,360, $4,684)
DCG_SKN_Infections_02	1,397,997	$933	($912, $954)
DCG_SKN_Infections_03	447,886	$172	($140, $204)
DCG_SKN_Infections_04	123,407	$172	($140, $204)
DCG_SKN_Cutaneous_structures_01	340,809	$1,367	($1,326, $1,408)
DCG_SKN_Cutaneous_structures_02	12,768	$1,193	($984, $1,402)
DCG_SKN_Cutaneous_structures_03	407,016	$735	($698, $772)
DCG_SKN_Cutaneous_structures_04	1,119,420	$191	($168, $214)
DCG_SKN_Cutaneous_structures_05	43,350	$191	($168, $214)
DCG_SKN_Allergies_01	5,418	$4,953	($4,632, $5,273)
DCG_SKN_Allergies_02	53,575	$822	($616, $1,027)
DCG_SKN_Allergies_04	128,314	$141	($75, $207)
DCG_SKN_External_factors_01	7,863	$13,159	($12,890, $13,427)
DCG_SKN_Intraop_postproc_01	19,630	$15,178	($15,008, $15,348)
DCG_SKN_Symptoms_01	3,507	$9,765	($9,366, $10,164)
DCG_SKN_Symptoms_02	272,689	$1,383	($1,338, $1,429)
DCG_SKN_Symptoms_03	430,372	$614	($577, $651)
DCG_SKN_Symptoms_04	549,454	$264	($232, $296)
DCG_SYM_Symptoms_01	23,412	$16,915	($16,721, $17,110)
DCG_SYM_Symptoms_02	4,835	$14,353	($14,011, $14,696)
DCG_SYM_Symptoms_03	5,476	$8,766	($8,446, $9,085)
DCG_SYM_Symptoms_04	204,217	$3,522	($3,467, $3,576)
DCG_SYM_Symptoms_05	1,202,909	$2,009	($1,987, $2,032)
DCG_SYM_Symptoms_06	6,710,322	$553	($542, $564)

Notes: DCG is the Diagnostic Cost Group algorithm; BLD is Blood and blood-forming organs and certain disorders involving the immune mechanism; CIR is Circulatory system; DIG is Digestive system; EAR is Ear and mastoid process; END is Endocrine, nutritional, and metabolic diseases; EYE is eye and adnexa; GEN is genitourinary system; INF is Certain infectious and parasitic diseases; INJ is Injury, poisoning, and certain other consequences of external causes, MAL is Congenital malformations, deformations and chromosomal abnormalities; MBD is Mental and behavioral disorders; MSK is musculoskeletal system and connective tissue; NEO is Neoplasms; NVS is Nervous system; PNL is Certain conditions originating in the perinatal period; PRG is Pregnancy, childbirth, and the puerperium; RSP is Respiratory system; SKN is Skin and subcutaneous tissue; and SYM is Symptoms, signs, and abnormal clinical and laboratory findings, not elsewhere classified.

© 2024 Andriola C et al. *JAMA Health Forum*.

eReferences.

[1] Rose S. A machine learning framework for plan payment risk adjustment. *Health Services Research*. 2016; 51(6).

[2] Ellis RP, Hsu HE, Siracuse JJ, Walkey AJ, Lasser KE, Jacobson BC, Andriola C, Hoagland A, Liu Y, Song C, Kuo TC. Development and Assessment of a New Framework for Disease Surveillance, Prediction, and Risk Adjustment: The Diagnostic Items Classification System. *JAMA Health Forum*. 2022; 3(3).

[3] Park S, Basu A. Improving risk adjustment with machine learning: accounting for service-level propensity scores to reduce service-level selection. *Health Services and Outcomes Research Methodology*. 2021; 21(3).

[4] Ash A, Porell F, Gruenberg L, Sawitz E, Beiser A. Adjusting Medicare capitation payments using prior hospitalization data. *Health Care Financing Review*. 1989;10(4).

[5] Pope GC, Ellis RP, Ash AS, Liu CF, Ayanian JZ, Bates DW, Burstin H, Iezzoni LI, Ingber MJ. Principal inpatient diagnostic cost group model for Medicare risk adjustment. *Health Care Financing Review*. 2000b; 21(3).

[6] Ash AS, Ellis RP, Pope GC, Ayanian JZ, Bates DW, Burstin H, Iezzoni LI, MacKay E, Yu W. Using diagnoses to describe populations and predict costs. *Health Care Financing Review*. 2000; 21(3).

© 2024 Andriola C et al. *JAMA Health Forum*.
