# Supplementary material for: A Novel Machine Learning Algorithm for Creating Risk-Adjusted Payment Formulas
Source: JAMA Health Forum. 2024 Apr 19;5(4):e240625. doi: 10.1001/jamahealthforum.2024.0625 (PMC11065160; doi:10.1001/jamahealthforum.2024.0625)
Supplement: Supplement 2. — Data Sharing Statement [file jamahealthforum-e240625-s002.pdf]

Data Sharing Statement

Andriola. A Novel Machine Learning Algorithm for Creating Risk-Adjusted Payment Formulas. *JAMA Health Forum*. Published April 19, 2024. doi:10.1001/jamahealthforum.2024.0625

Data
Data available: No

Additional Information
Explanation for why data not available: While we are unable to share the detailed claims and enrollment data or analytical files due to licensing restrictions on the Marketscan data, the same data can be licensed and is in use by other researchers. We intend to post the base model DCG mappings hierarchies, ATI scores, and software details that would enable replication and application of the algorithm on the same or different datasets.
